# ﻿Unveiling species diversity within early-diverging fungi from China III: Six new species and a new record of *Gongronella* (Cunninghamellaceae, Mucoromycota)

**DOI:** 10.3897/mycokeys.110.130260

**Published:** 2024-11-20

**Authors:** Yi-Xin Wang, Heng Zhao, Yang Jiang, Xin-Ye Liu, Meng-Fei Tao, Xiao-Yong Liu

**Affiliations:** 1 College of Life Sciences, Shandong Normal University, Jinan 250358, China Shandong Normal University Jinan China; 2 School of Ecology and Nature Conservation, Beijing Forestry University, Beijing 100083, China Beijing Forestry University Beijing China; 3 State Key Laboratory of Mycology, Institute of Microbiology, Chinese Academy of Sciences, Beijing 100101, China Institute of Microbiology, Chinese Academy of Sciences Beijing China

**Keywords:** Mucoromycota, molecular phylogeny, new taxa, soil-borne fungi, taxonomy

## Abstract

*Gongronella* had accommodated only two species for more than half a century and as many as 17 new species have been described in this genus since 2015. However, no systematic studies were conducted for this genus so far. The distribution, substrate and morphology of all known species in *Gongronella* are analysed herein. Meanwhile, with the support of phylogenetic and morphological evidence, six new species (*G.abortosporangia***sp. nov.**, *G.apophysata***sp. nov.**, *G.bawanglingensis***sp. nov.**, *G.inconstans***sp. nov.**, *G.pingtangensis***sp. nov.** and *G.reniformis***sp. nov.**) are proposed and *G.pamphilae* is recorded from China for the first time. The phylogenetic tree was constructed using ITS+LSU+TEF+ACT+RPB1 and the results were basically the same as ITS+LSU. All species of *Gongronella*, except *G.namwonensis* from fresh water, were isolated from soil. The genus is distributed worldwide, mainly in tropical and subtropical regions. A synoptic key is provided for a total of 24 species (18 species previously published and six species newly described herein), except for *G.banzhaoae* due to unavailable protologue, type and living culture. No morphologies were described when *G.pamphilae* was proposed. Thanks to the strains isolated in this study, *G.pamphilae* is included in the key and reported as a Chinese new record. This is the first comprehensive taxonomy and phylogeny of the genus *Gongronella*.

## ﻿Introduction

The genus *Gongronella* Ribaldi has a great potential in biological applications due to the ability of producing bioactive substance such as chitosan (Wang et al. 2008; Zhou et al. 2008), dissolving phosphate and degrading metalaxyl (Doilom et al. 2020; Martins et al. 2020). *Gongronella* sp. w5, a well-known strain in this genus, can induce fungi *Panusrudis* (Wei et al. 2010) and *Coprinopsiscinerea* (Pan et al. 2014; Hu et al. 2019; Liu et al. 2022) to produce laccase, secrete organic acids for improving the acquisition of phosphate in plants and thus promote their growth (Dong et al. 2018; Wang et al. 2021) and synthesise various bioactive enzymes, such as β-glucosidase and invertase (Zhou et al. 2020; Mai et al. 2021).

This genus was established in 1952 and typified with *Gongronellaurceolifera* Ribaldi (Ribaldi 1952). It belongs to Mucoromycota Doweld, Mucoromycetes Doweld, *Mucorales* Dumort, Cunninghamellaceae Naumov ex R.K. Benj. (Tedersoo et al. 2018). Before 2015, the taxonomy of *Gongronella* was stagnant, accommodating only two species *G.urceolifera* (= *G.butleri*) and *G.lacrispora*. Since 2015, as many as 17 species have been described successively (Hesseltine and Ellis 1961; Adamcik et al. 2015; Ariyawansa et al. 2015; Li et al. 2016; Tibpromma et al. 2017; Dong et al. 2019; Zhang et al. 2019; Crous et al. 2020; de Freitas et al. 2020; Doilom et al. 2020; Martins et al. 2020; Wang et al. 2023a; Zhao et al. 2023). At present, *Gongronella* contains 19 species, nearly half of which were initially found from China (Table 1). In the GlobalFungi database, there are a total of 3,039 sample records for the genus *Gongronella* covering Asia (1,566, 51.53%), North America (571, 18.79%), Europe (433, 14.58%), South America (261, 8.59%), Africa (123, 4.05%), Australia (64, 2.11%) and Atlantic Ocean (1, 0.03%) (https://globalfungi.com/, accessed on 18 October 2024). Considering geographical climate, most samples were collected from tropical and subtropical regions (https://globalfungi.com/, accessed on 17 October 2024). In conclusion, the species of *Gongronella* were distributed worldwide and mainly concentrated in tropical and subtropical regions in Asia.

**Table 1.** The origin of taxonomic types in *Gongronella*.

Countries	Type numbers	Percentage (%)
China	9	47.4
Korea	3	15.8
Brazil	3	15.8
Australia	2	10.5
Portugal	1	5.3
UK	1	5.3

Note: These data are from the Index Fungorum (http://www.indexfungorum.org/, accessed on 9 December 2023) and Wang et al. (2023).

Regarding substrate of nomenclatural types within the genus *Gongronella*, *G.namwonensis* was isolated from fresh water and the other 18 species were all isolated from soil (Crous et al. 2020; Doilom et al. 2020). According to the GlobalFungi database, substrates include soil (1852, 60.94%), topsoil (475, 15.63%), root (403, 13.26%), rhizosphere soil (204, 6.71%), root + rhizosphere soil (52, 1.71%), litter (22, 0.72%), sediment (10, 0.33%), shoot (9, 0.3%) and deadwood (7, 0.23%), (https://globalfungi.com/, accessed on 19 October 2024). Although the GlobalFungi database showed more kinds of substrates of *Gongronella*, most strains were still isolated from a variety of soil samples.

In this study, 14 strains of the genus *Gongronella* were isolated from soil in Hainan, Yunnan, Sichuan and Guizhou Provinces from China. According to ITS+LSU+TEF+RPB1 molecular phylogenetic analyses and morphological comparisons, these strains were classified into six new species and one was identified as new record species to China. The morphological information of all described species of *Gongronella* was reviewed and compared.

## ﻿Materials and methods

### ﻿Isolation and morphology

Soil samples were collected in Hainan Province (April 2023 and October 2023), Sichuan Province (June 2023) and Guizhou Province (August 2023). Strains were isolated from the soil samples by a combination of soil dilution and single spore isolation.

About 1 g soil sample was mixed with 10 ml sterile water to prepare 10^-1^ soil suspension. One millilitre of 10^-1^ suspension was transferred to 9 ml of sterile water to obtain a 10^-2^ soil suspension. In the same way, 10^-3^ and 10^-4^ soil suspensions were made. The final 10^-3^ and 10^-4^ soil suspensions (200 ml) were pipetted on the surface of Rose-Bengal Chloramphenicol Agar (RBC: peptone 5.00 g/l, glucose 10.00 g/l, KH_2_PO_4_ 1.00 g/l, MgSO_4_·7H_2_O 0.50 g/l, rose red 0.05 g/l, chloramphenicol 0.10 g/l, agar 15.00 g/l) (Corry et al. 1995), dispersed evenly with sterilised coating rods and cultured at 25 °C in the dark for 2–5 days. Upon colonies were visible, they were transferred onto Potato Dextrose Agar (PDA: glucose 20.00 g/l, potato 200.00 g/l, agar 20.00 g/l, pH 7). When sporangia were produced, sporangiospores were suspended with sterile water and streaked with a sterilised inoculation ring. The plates were cultured at 25 °C in darkness and single spore colonies were transferred on to a new PDA plate for subculturing. To ensure the formation of zygospores, pairing experiments were carried out by adding 0.1% lecithin to PDA and sealing Petri dishes to retain moisture. The microscopic morphological characteristics of fungi were observed with an optical microscope (Olympus BX53) and photographed with a high-definition colour digital camera (Olympus DP80). All strains were stored with 10% sterilised glycerine at 4 °C. Each morphological character was statistically calculated from 30 measurements (Zhang et al. 2022). Cultures were deposited in the China General Microbiological Culture Collection Center, Beijing, China (CGMCC) and the Shandong Agricultural University Culture Collection, Taian, China (SAUCC). Specimens were deposited in the Herbarium Mycologicum Academiae Sinicae, Beijing, China (HMAS). Taxonomic information for the new taxa was registered in the Fungal Name repository (https://nmdc.cn/fungalnames/).

### ﻿DNA extraction and amplification

Genomic DNA was extracted from mycelia using the CTAB method and BeaverBeads Plant DNA Kit (Cat. No.: 70409-20; BEAVER Biomedical Engineering Co., Ltd.) (Doyle et al. 1990; Guo et al. 2000; Wang et al. 2023b). ITS, LSU, TEF, ACT and RPB1 were amplified by polymerase chain reaction using ITS4/ITS5, LR0R/LR7, EF1-728F/EF1-986R, ACT-512F/ACT-783R and RPB1-Af/RPB1-Cr primer pairs, respectively (Table 2). Amplification was performed in a final volume of 20 μl, containing 10 μl 2× Hieff Canace^®^ Plus PCR Master Mix (Yeasen Biotechnology, Cat No. 10154ES03), 0.5 μl of forward and reverse primers each (10 μM) (TsingKe, Beijing, China), 1 μl template genomic DNA (about 1 μM) and 8 μl distilled deionised water. Molecular loci, PCR primers and programmes used in this study are listed in Table 2. The PCR products were electrophoresed with 1% agarose gel. The DNA fragments were stained with GelRed and observed under ultraviolet light. Then a gel extraction kit (Cat# AE0101-C; Shandong Sparkiade Biotechnology Co., Ltd.) was used for gel recovery. Sanger sequencing was carried out by Biosune Company Limited (Shanghai, China). Consensus sequences were assembled using MEGA v.7.0 (Kumar et al. 2016). All sequences generated in this study were deposited at GenBank under the accession numbers in Table 3.

**Table 2.** Molecular loci, PCR primers and programmes used in this study.

Loci	PCR primers	Sequence (5’–3’)	PCR cycles	References
ITS	ITS5	GGA AGT AAA AGT CGT AAC AAG G	95 °C 5 min; (95 °C 30 s, 55 °C 30 s, 72 °C 1 min) × 35 cycles; 72 °C 10 min	White et al. (1990)
	ITS4	TCC TCC GCT TAT TGA TAT GC		
LSU	LR0R	GTA CCC GCT GAA CTT AAG C	95 °C 5 min; (95 °C 50 s, 47 °C 30 s, 72 °C 1.5 min) × 35 cycles; 72 °C 10 min	Vilgalys and Hester (1990)
	LR7	TAC TAC CAC CAA GAT CT		
TEF	EF1-728F	CAT CGA GAA GTT CGA GAA GG	95 °C 5 min; (95 °C 30 s, 55 °C 60 s, 72 °C 1 min) × 30 cycles; 72 °C 10 min	Carbone and Kohn (1999); O’Donnell et al. (1998)
	EF2	GGA RGT ACC AGT SAT CAT GTT		
RPB1	RPB1-Af	GAR TGY CCD GGD CAY TTY GG	95 °C 3 min; (94 °C 40 s, 60 °C 40 s, 72 °C 2 min) × 9 (94 °C 45 s, 55 °C 1.5 min, 72 °C 2 min) × 37 cycles; 72 °C 10 min	Stiller and Hall (1997)
	RPB1-Cr	CCN GCD ATN TCR TTR TCC ATR TA		
ACT	ACT-512F	ATG TGC AAG GCC GGT TTC GC	95 °C 3 min; (95 °C 1 min, 55 °C 1 min, 72 °C 1 min) × 30 cycles; 72 °C 10 min	Voigt and Wostemeyer (2000)
	ACT-783R	TAC GAG TCC TTC TGG CCC AT		

**Table 3.** Information of strains used in this study.

Species	Strains	Substrates	Countries	GenBank accession numbers				
				ITS	LSU	ACT	TEF	RPB1
***Gongronellaabortosporangia***	CGMCC 3.27028*	**Soil**	**China**	**PP195847**	**PP195948**	**PP933938**	**PP850088**	**PP842883**
	**SAUCC 4064-2**	**Soil**	**China**	**PP195848**	**PP195949**	**PP933939**	**PP850089**	**PP842882**
***G.apophysata***	CGMCC 3.27031*	**Soil**	**China**	**PP195853**	**PP195954**	**PP933947**	PP850099	**PP842878**
	**SAUCC 4846-3**	**Soil**	**China**	**PP195854**	PP195955	**PP933948**	**PP850100**	**PP842877**
*G.banzhaoae*	BRIP 75171a*	Soil	Australia	OR271908	OR259049	n.a.	n.a.	n.a.
***G.bawanglingensis***	CGMCC 3.27033*	**Soil**	**China**	**PP195857**	**PP195958**	**PP933951**	**PP850103**	**PP883965**
	**SAUCC 6946-1**	**Soil**	**China**	**PP195858**	**PP195959**	**PP933952**	**PP850104**	**PP883964**
*G.brasiliensis*	URM 7487*	Soil	Brazil	NR_155148	KY114932	n.a.	n.a.	n.a.
	URM 7488	Soil	Brazil	KY114931	KY114933	n.a.	n.a.	n.a.
*G.butleri*	CBS 216.58*	Soil	UK	JN206285	MH869292	n.a.	n.a.	n.a.
*G.chlamydospora*	CGMCC 3.16118*	Soil	China	OL678157	n.a.	n.a.	n.a.	PP898292
*G.eborensis*	MUM 10.262*	Soil	Portugal	KT809408	MN947301	n.a.	n.a.	n.a.
	MUM 10.263	Soil	Portugal	GU244500	MN947302	n.a.	n.a.	n.a.
*G.guangdongensis*	CGMCC 2.15212*	Soil	China	NR_158464	MN947303	n.a.	n.a.	n.a.
	CGMCC 2.15213	Soil	China	KC462740	MN947304	n.a.	n.a.	n.a.
*G.hydei*	KUMCC 18.0198*	Rhizosphere soil	China	NR_171964	MT907273	n.a.	n.a.	n.a.
***G.inconstans***	CGMCC 3.27029*	**Soil**	**China**	**PP195849**	**PP195950**	**PP933941**	**PP850091**	**PP842874**
	**SAUCC 4113-3**	**Soil**	**China**	**PP195850**	**PP195951**	**PP933942**	**PP850092**	**PP842873**
*G.koreana*	EML-TS2Bp*	Soil	Korea	KP636529	KP636530	KP636527	n.a.	n.a.
	EML-TS2Bp-2	Soil	Korea	KP835545	KP835542	KP835543	n.a.	n.a.
*G.lacrispora*	ATCC 24412*	Soil	Brazil	GU244498	JN206609	n.a.	n.a.	n.a.
*G.multiramosa*	CGMCC 3.26216*	Soil	China	OR733546	OR733611	PP933937	PP850087	PP842881
	SAUCC 4056-4	Soil	China	OR733545	OR733610	n.a.	n.a.	n.a.
*G.multispora*	CGMCC 3.16119*	Soil	China	OL678158	n.a.	n.a.	n.a.	pm
*G.namwonensis*	CNUFC WW2-12*	Fresh water	Korea	NR_175640	MN658482	n.a.	n.a.	n.a.
*G.oleae*	CGMCC 3.26217*	Soil	China	OR742078	OR733608	PP933945	PP850097	PP850080
	SAUCC 4164-2	Soil	China	OR742079	OR733609	PP933946	PP850098	PP850079
*G.orasabula*	EML-QF12-1*	Soil	Korea	NR_148087	KT936263	KT936265	n.a.	n.a.
	EML-QF12-2	Soil	Korea	KT936270	KT936264	n.a.	n.a.	n.a.
*G.pamphilae*	BRIP 74936a*	Soil	Australia	OR271909	OR259050	n.a.	n.a.	n.a.
	**CGMCC 3.27027**	**Soil**	**China**	**PP195845**	**PP195946**	**PP933935**	**PP850086**	**PP850081**
	**SAUCC 4031-2**	**Soil**	**China**	**PP195846**	**PP195947**	**PP933936**	**PP850085**	**PP850082**
*G.pedratalhadensis*	URM 8182*	Soil	Brazil	MN912512	MN912508	n.a.	n.a.	n.a.
***G.pingtangensis***	CGMCC 3.27032*	**Soil**	**China**	**PP195855**	**PP195956**	**PP933949**	**PP850101**	**PP842880**
	**SAUCC 5676-2**	**Soil**	**China**	**PP195856**	**PP195957**	**PP933950**	**PP850102**	**PP842879**
*G.qichaensis*	CGMCC 3.26218*	Soil	China	OR733544	OR733607	n.a.	**PP850093**	**PP850084**
	SAUCC 4137-3	Soil	China	OR733543	OR733606	n.a.	**PP850094**	**PP850083**
***G.reniformis***	CGMCC 3.27030*	**Soil**	**China**	**PP195851**	**PP195952**	**PP933943**	**PP850095**	**PP842875**
	**SAUCC 4142-5**	**Soil**	**China**	**PP195852**	**PP195953**	**PP933944**	**PP850096**	**PP842876**
*G.sichuanensis*	CGMCC 3.19651*	Soil	China	MK813373	MK813855	MK820625	n.a.	n.a.
	CGMCC 3.19652	Soil	China	MK813374	MK813856	MK820626	n.a.	n.a.
*G.zunyiensis*	CGMCC 3.19899*	Soil	China	MN453856	MN453853	n.a.	n.a.	n.a.
	CGMCC 3.19900	Soil	China	MN453857	MN453854	n.a.	n.a.	n.a.
*Cunninghamellaechinulata*	CBS 156.28*	n.a.	n.a.	JN205895	MH877699	n.a.	n.a.	n.a.

Notes: New species established in this study are in bold. Ex-type or ex-holotype strains are labelled with a star mark “*”. The abbreviation “n.a.” stands for “not available”

Relative sequences were obtained by BLAST search in the GenBank nucleotide database of NCBI website (Kumar et al. 2016). Sequences both generated herein and retrieved from GenBank (Table 3) were aligned using MAFFT 7 online service (http://mafft.cbrc.jp/alignment/server/, 20 October 2023) (Katoh et al. 2019). The ITS, LSU, TEF, ACT and RPB1 sequences were analysed individually and jointly. The optimal evolutionary model for each partition was determined and included in the analysis using MrModelTest v.2.3 (Nylander 2004). Phylogenetic history was reconstructed using Maximum Likelihood (ML) algorithm with RaxML-HPC2 on XSEDE 8.2.12 (Stamatakis 2014; Zhao et al. 2024) and Bayesian Inference (BI) algorithm with MrBayes 3.2.7a (Huelsenbeck and Ronquist 2001; Ronquist and Huelsenbeck 2003; Ronquist et al. 2012). ML was performed for 1,000 bootstrap replicates with the GTRGAMMA model of nucleotide evolution. BI was performed using a quick start algorithm with an automatic stop option. The Bayesian analysis consisted of 5,000,000 generations with four parallel runs with the option of stopping rules and a sampling frequency of 100 generations. The burn-in score was set to 0.25 and the posterior probability (PP) was determined from the remaining trees. Initial adjustments of phylogenetic trees were made using FigTree v.1.4.4 (http://tree.bio.ed.ac) and the layout of the trees was finished using Adobe Illustrator CC 2019 (https://adobe.com/products/illustrator).

## ﻿Results

### ﻿Phylogenetic analyses

**Figure 1.**
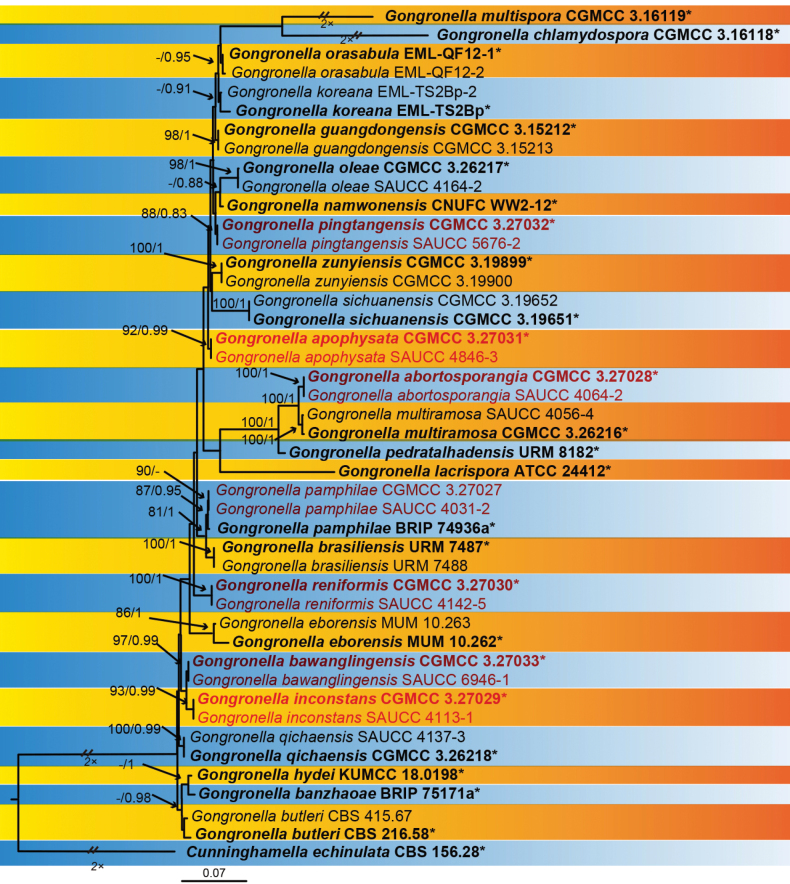
A Maximum Likelihood (ML) phylogenetic consensus tree inferred from DNA sequences of ITS, LSU, TEF, ACT and RPB1, showing relationships amongst species of *Gongronella* with *Cunninghamellaechinulata* CBS 156.28 as outgroup. The Maximum Likelihood bootstrap value (MLBV) and Bayesian Inference posterior probability (BIPP) are successively shown at the nodes and separated by a slash “/”. Strains marked with a star “*” are ex-types or ex-holotypes. The strains isolated and sequenced in this study are shown in red. Branches shortened to fit the page are represented by double slashes “//” and folds “×”. The scale in the bottom centre indicates 0.2 substitutions per site.

The sequence matrix included 43 strains in 25 species of *Gongronella*, with *Cunninghamellaechinulata* CBS 156.28 as outgroup. A total of 4,080 characters comprised ITS rDNA (1–989), LSU rDNA (990–1967), TEF (1968–2172), ACT (2173–2948) and RPB1 (2949–4080). Amongst these characters, 2,866 were constant, 562 variable, but parsimony non-informative and 652 parsimony informative characters (Suppl. material 1). MrModelTest suggested that the Dirichlet fundamental frequency and GTR+I+G evolution pattern for both partitions were adopted in Bayesian Inference. The topology of the Bayesian tree was consistent with that of the ML tree and, therefore, was used as a representative to summarise the evolutionary history within the genus *Gongronella* (Fig. 1). *G.abortosporangia* was closely related to *G.multiramosa* with a high support (BIPP = 0.95). *G.pingtangensis* was closely related to *G.namwonensis* with a high support (BIPP = 1). *G.reniformis* was closely related to *G.pamphilae* and *G.brasiliensis* with a high support (MLBV = 75, BIPP = 0.99). The *G.bawanglingensis* (MLBV = 100, BIPP = 1) is closely related to *G.qichaensis* and *G.inconstans*. *G.inconstans* (MLBV = 99, BIPP = 1) is closely related to *G.qichaensis* with a high support (BIPP = 0.96). *G.apophysata* is closely related to *G.zunyiensis*.

### ﻿Taxonomy

#### ﻿ Gongronella abortosporangia

Taxon classificationFungiMucoralesCunninghamellaceae

Yi Xin Wang, H. Zhao & X.Y. Liu sp. nov.

21D8BDF9-EF04-5AF3-A57F-BA9041EF991A

Fungal Names: FN 571253

Fig. 2

##### Etymology.

The epithet “*abortosporangia*” (Latin) refers to the abortive sporangia.

##### Type.

China • Hainan Province, Lingshui Li Autonomous County, Qixian Yaochi Yexi Hot Spring (18.70161°N, 109.69318°E), from soil sample, 10 April 2023, Yi-Xin Wang (holotype HMAS 352726, ex-holotype strain CGMCC 3.27028).

##### Description.

Colonies growing slowly on PDA in darkness at 25 °C, reaching 49.2–52.4 mm in diameter in seven days, white, regular at edge and cottony in the centre, reversely milky white. Rhizoids hyaline, branched, irregularly shaped, with oil droplets. Stolons absent. Sporangiophores on aerial mycelia, erect or slightly curved, unbranched or branched (1–6 times), 4.0–96.8 × 1.0–4.2 μm, hyaline, smooth, mostly aseptate, sometimes one-septate and rarely two-septate, occasionally containing a line of oil droplets. Sterile (aborted) sporangia abundant, mainly on the top of short lateral branches of sporangiophores, mostly gourd-shaped, 11.6–16.7 × 5.5–17.7 μm, partially elliptical with a slight shrinkage, 12.5–18.0 × 6.7–10.6 μm, occasionally clavate, 20.1–22.7 × 9.5–10.4 μm. Fertile sporangia hyaline or light yellow, spherical, 7.0–23.2 μm in diameter, smooth and deliquescent-walled, leaving a collar after releasing sporangiospores. Columellae mostly hemispherical, 2.5–4.2 × 3.6–7.4 μm, sometimes sub-hemispherical, 1.3–3.9 × 3.6–5.5 μm, hyaline, smooth. Apophyses hyaline, smooth, variously shaped, mostly cup-shaped, 1.9–8.6 × 2.1–6.7 μm, partially hemispherical, 2.7–5.5 × 2.8–7.4 μm, occasionally pear-shaped, 8.2 × 7.2 μm. Sporangiospores not uniform, hyaline, smooth, ovoid, 2.6–3.5 × 1.7–2.1 μm, reniform, 2.9–3.5 × 1.7–2.3 μm. Chlamydospores gourd-shaped, 20.3–29.3 × 6.4–9.3 μm. Giant cells intercalary, globular, subglobular, 2.6–4.6 μm in diameter. Zygospores not found.

**Figure 2.**
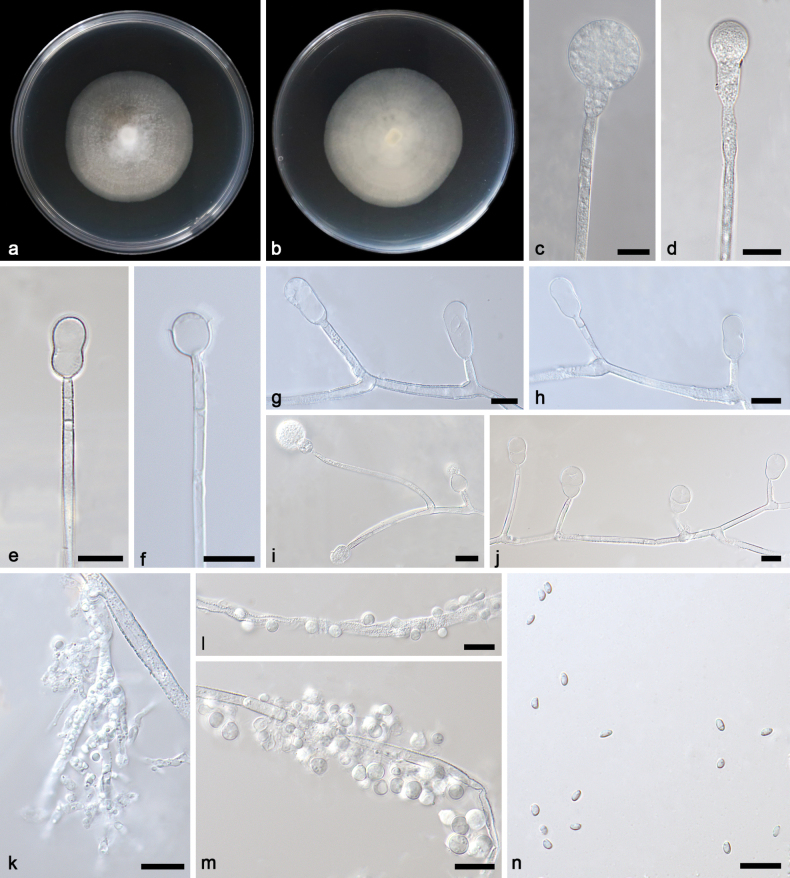
*Gongronellaabortosporangia* ex-holotype CGMCC 3.27028 **a**, **b** colonies on PDA (**a** obverse **b** reverse) **c** an unbranched sporangiophore with a mature sporangium **d** an unbranched sporangiophore with an immature sporangium **e** an aborted sporangium with two septa **f** columellae, collars and apophyses **g**, **h**, **j** branched sporangiophores with sterile (aborted) sporangia **i** a branched sporangiophore with a mature sporangium, columellae, collars and apophyses **k** rhizoids **l**, **m** giant cells **n** sporangiospores. Scale bars: 10 μm (**c–n**).

##### Additional specimen examined.

China • Hainan Province, Lingshui Li Autonomous County, Benhao Town (18.70161°N, 109.69318°E), from soil sample, 10 April 2023, Yi-Xin Wang (living culture SAUCC 4064-2).

##### GenBank accession numbers.

CGMCC 327028 (ITS, PP195847; LSU, PP195948; TEF, PP850088; ACT, PP933938; RPB1, PP842883), SAUCC 4064-2 (ITS, PP195848; LSU, PP195949; TEF, PP850089; ACT, PP933939; RPB1, PP842882).

##### Notes.

Based on phylogenetic analyses of ITS+LSU+TEF+ACT+RPB1 sequences, the two isolates of the new species *Gongronellaabortosporangia* formed an independent clade with high supports (MLBV = 100; Fig. 1), which is closely related to *G.multiramosa* (BIPP = 0.95; Fig. 1). This new species differs morphologically from *G.multiramosa* in sporangium, septum, columella, collar and apophysis (Wang et al. 2023a). The *G.abortosporangia* is different from *G.multiramosa* in shape and size of sterile sporangia, the former being variously shaped, mostly gourd-shaped, 11.6–16.7 × 5.5–17.7 μm, partially elliptical with a slight shrinkage, 12.5–18.0 × 6.7–10.6 μm, occasionally clavate, 20.1–22.7 × 9.5–10.4 μm, while the latter being only ovoid, 9.6 × 6.2 µm in diameter. In fertile sporangia, *G.abortosporangia* has a smaller minimum diameter than *G.multiramosa* (7.0 μm vs. 15.5 μm). *G.abortosporangia* has more septa on sporangiophores compared to *G.multiramosa* (0–2 vs. 0–1). Although *G.abortosporangia* is similar in shape of columellae to *G.multiramosa*, it is shorter in length (hemispherical, 3.6–7.4 µm vs. 8.0–9.8 µm, sub-hemispherical, 3.6–5.5 μm vs. 7.6–10.0 µm). The *G.abortosporangia* has shorter collars than *G.multiramosa*, 0.6–3.9 μm vs. 1.3–7.2 µm. The *G.abortosporangia* is similar in shape of apophyses to *G.multiramosa*. However, they are different from each other in main pattern and size: The former mostly cup-shaped (1.9–8.6 × 2.1–6.7 μm vs. 4.6–7.0 × 8.5–10.0 µm) and partially hemispherical (2.7–5.5 × 2.8–7.4 μm vs. 4.4–5.6 × 8.5–9.0 µm) and the latter opposite. Combining morphological and molecular phylogenetic analyses, we classified the two isolates as a new species *G.abortosporangia* allied to *G.multiramosa*.

#### ﻿ Gongronella apophysata

Taxon classificationFungiMucoralesCunninghamellaceae

Yi Xin Wang, H. Zhao & X.Y. Liu sp. nov.

7FE20022-07AB-54AB-9323-FBC903DC5A81

Fungal Names: FN 571631

Fig. 3

##### Etymology.

The epithet “*apophysata*” (Latin) refers to various shapes of apophyses.

##### Type.

China • Sichuan Province, Emeishan City, Leshan City, Ehong Road, near the Xu family residence (29.59211°N, 103.37776°E), from soil sample, 25 June 2023, Yi-Xin Wang (holotype HMAS 352728, ex-holotype strain CGMCC 3.27031).

##### Description.

Colonies growing slowly on PDA in darkness at 25 °C, reaching 35.8–42.4 mm in diameter in seven days, white, irregular at edge and cottony in the centrr, reversely milky white. Rhizoids hyaline, branched, irregularly shaped. Stolons absent. Sporangiophores on aerial mycelia, erect or slightly curved, unbranched or slightly branched (1–2 times), 11.2–190.9 × 1.6–3.9 μm, hyaline, smooth, mostly aseptate or one-septate, occasionally two-septate. Sterile (aborted) sporangia predominantly on the top of short lateral branches of sporangiophores, gourd-shaped, 14.0 × 8.3 μm. Fertile sporangia hyaline or light yellow, spherical, 12.5–40.5 μm in diameter, smooth and deliquescent-walled, leaving a collar after releasing sporangiospores. Columellae elliptic, 2.6–4.0 × 2.1–5.5 μm, sub-hemispherical, 1.4–2.7 × 2.2–4.3 μm, hyaline, smooth. Apophyses hyaline, smooth, variously shaped, mostly ellipsoidal to olive-shaped, 2.3–17.3 × 2.4–10.0 μm, partially subglobose, 4.6–10.2 × 4.3–10.0 μm, occasionally gourd-shaped, 11.4 × 4.9 μm. Sporangiospores not uniform, hyaline, smooth, mostly reniform, 3.2–5.5 × 1.7–3.1 μm, ovoid, 2.5–3.7 × 1.7–2.6 μm, occasionally subglobose, 1.7–2.5 μm. Chlamydospores present, not uniform, gourd-shaped, ellipsoidal and suborbicular, mostly gourd-shaped, 23.5–35.4 × 10.8–14.0 μm, partially ellipsoidal, 18.6–21.4 × 10.3–18.5 μm. Giant cells in the rhizoids, intercalary, globose, 4.4–10.5 μm in diameter. Zygospores not found.

**Figure 3.**
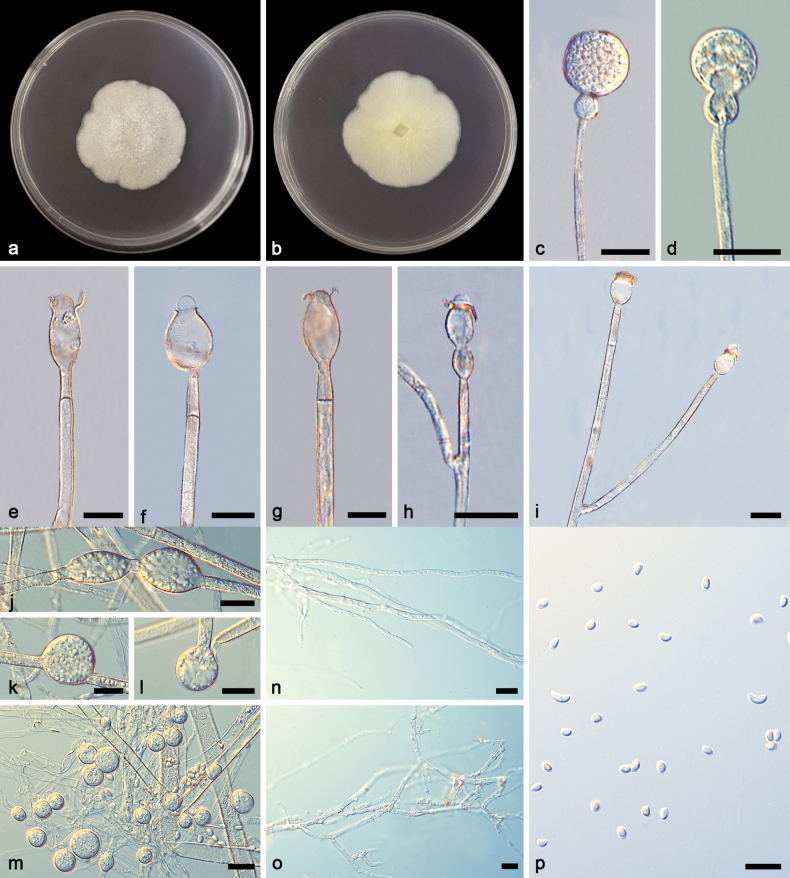
*Gongronellaapophysata* ex-holotype CGMCC 3.27031 **a**, **b** colonies on PDA (**a** obverse **b** reverse) **c** an unbranched sporangiophore with a fertile sporangium **d** an unbranched sporangiophore with an immature sporangium **e–g** columellae, collars, apophyses and septa **h** branched sporangiophores with columellae, collars and apophyses **i** branched sporangiophores with columellae, collars and apophyses **j–l** chlamydospores **m** giant cells **n**, **o** rhizoids **p** sporangiospores. Scale bars: 10 μm (**c–p**).

##### Additional specimen examined.

China • Sichuan Province, Emeishan City, Leshan City, Ehong Road, near the Xu family residence (29.59211°N, 103.37776°E), from soil sample, 25 June 2023, Yi-Xin Wang (living culture SAUCC 4846-3).

##### GenBank accession numbers.

CGMCC 3.27031 (ITS, PP195853; LSU, PP195954; TEF, PP850099; ACT, PP933947; RPB1, PP842878), SAUCC 4846-3 (ITS, PP195854; LSU, PP195956; TEF, PP850100; ACT, PP933948; RPB1, PP842877).

##### Notes.

Based on phylogenetic analyses of ITS+LSU+TEF+ACT+RPB1 sequences, the two isolates of the new species *Gongronellaapophysata* form an independent clade with high support (MLBV = 98; Fig. 1), which is closely related to *G.zunyiensis*. In ITS, *G.apophysata* differs from the type species of *G.zunyiensis* by 13 base pairs. This new species differs morphologically from *G.zunyiensis* in sporangium, columellae, apophyses and chlamydospore (Dong et al. 2019). *G.apophysata* has larger sporangia than *G.zunyiensis* (12.5–40.5 μm vs. 11.0–19.5 μm). *G.apophysata* differs from *G.zunyiensis* in the shape of columellae, the former being elliptic and the latter being hemispherical and globose. As for apophyses, *G.apophysata* and *G.zunyiensis* are remarkably different in shape and size, the former variously shaped, mostly ellipsoidal to olivary, 2.3–17.3 × 2.4–10.0 μm, partially subglobose, 4.6–10.2 × 4.3–10.0 μm, occasionally gourd-shaped, 11.4 × 4.9 μm and the latter hemispherical, 1.5–3.5 × 1.0–3.0 μm. *G.apophysata* is remarkably different from *G.zunyiensis* in shape and size of chlamydospores, the former being not uniform, mostly gourd-shaped, 23.5–35.4 × 10.8–14.0 μm, partially ellipsoidal, 18.6–21.4 × 10.3–18.5 μm and the latter being terminal or lateral, globose or subglobose, 7.0–10.5 μm in diameter. Combining morphological and molecular phylogenetic analyses, we classified the two isolates together as a new species *G.apophysata* allied to *G.zunyiensis*.

#### ﻿ Gongronella bawanglingensis

Taxon classificationFungiMucoralesCunninghamellaceae

Yi Xin Wang, H. Zhao & X.Y. Liu sp. nov.

D23F69CC-3F5F-52A8-8049-F7DCD0A0CC78

Fungal Names: FN 571903

Fig. 4

##### Etymology.

The epithet “*bawanglingensis*” (Latin) refers to the location where the type was collected, Bawangling National Forest Park.

##### Type.

China • Hainan Province, Changjiang Li Autonomous County, Bawangling National Forest Park (19.08593°N, 109.12275°E), from soil sample, 14 October 2023, Yi-Xin Wang (holotype HMAS 352730, ex-holotype strain CGMCC 3.27033).

##### Description.

Colonies growing slowly on PDA in darkness at 25 °C, reaching 45.6–48.8 mm in diameter in seven days, white, cottony in the centre, on the reverse milky white. Rhizoids hyaline, branched, irregularly shaped. Stolons absent. Sporangiophores on aerial mycelia, erect or slightly curved, unbranched or slightly branched (up to 3 times), sympodially branched, 1.3–4.5 μm in width, hyaline, smooth, mostly aseptate or one-septate, no more than four-septate. Sterile (aborted) sporangia mainly on the top of short lateral branches of sporangiophores, mostly gourd-shaped. Fertile sporangia hyaline or light yellow, spherical, 4.2–18.5 μm in diameter, smooth and deliquescent-walled, leaving a collar after releasing sporangiospores. Columellae mostly hemispherical, 1.6–5.1 × 2.1–7.2 μm, sometimes arch-shaped, 1.4–3.7 × 2.6–8.8 μm, occasionally spherical, 2.3–6.1 × 2.5–8.1 μm, hyaline, smooth. Collars mostly distinct, 0.7–5.9 μm. Apophyses hyaline, smooth, variously shaped, mostly oval, 3.9–20.6 × 3.3–12.9 μm, sometimes subglobose, 4.8–12.2 × 4.7–12.3 μm, occasionally gourd-shaped. Sporangiospores not uniform, hyaline, smooth, mostly ovoid, 2.5–3.6 × 1.7–2.6 μm, partially reniform, 2.6–3.3 × 1.9–2.2 μm. Chlamydospores not uniform, gourd-shaped, 15.1–24.6 × 7.4–12.9 μm, ellipsoidal, 15.1–18.6 × 8.3–14.0 μm, suborbicular, 12.6–13.5 μm in diameter. Giant cells intercalary, globular, 3.2–6.9 μm in diameter. Zygospores not found.

**Figure 4.**
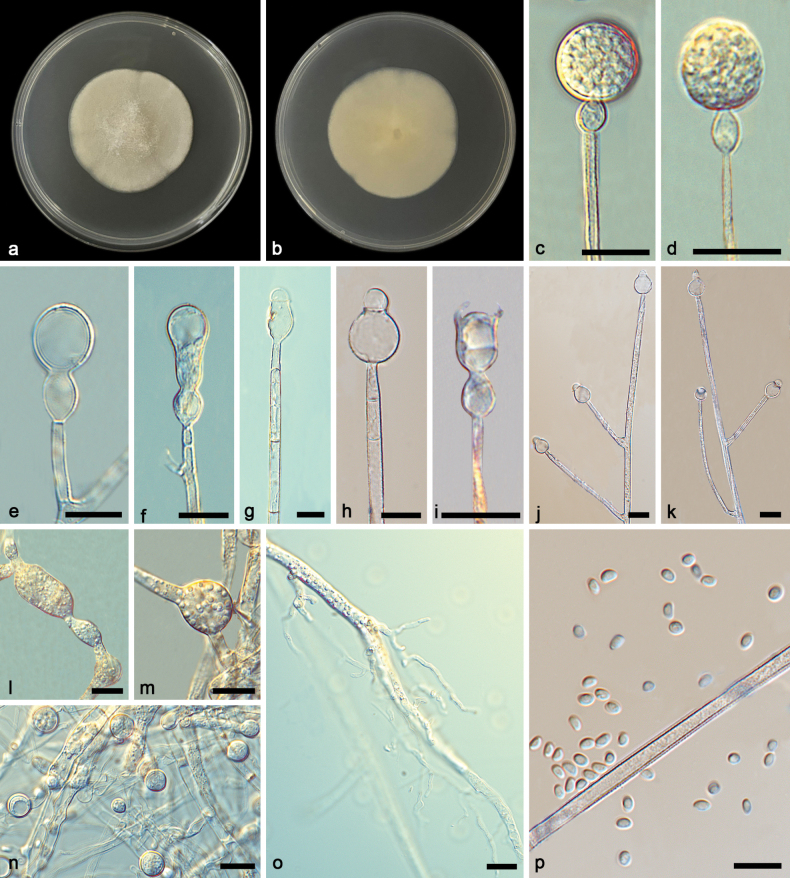
*Gongronellabawanglingensis* ex-holotype CGMCC 3.27033 **a**, **b** colonies on PDA (**a** obverse **b** reverse) **c**, **d** an unbranched sporangiophore with a fertile sporangium **e** branched sporangiophores with sterile (aborted) sporangia **f** branched sporangiophores with immature sporangia **g–i** columellae, collars, apophyses and septa **j**, **k** branched sporangiophores with columellae, collars and apophyses **l**, **m** chlamydospores **n** giant cells **o** rhizoids **p** sporangiospores. Scale bars: 10 μm (**c–p**).

##### Additional specimen examined.

China • Hainan Province, Changjiang Li Autonomous County, Bawangling National Forest Park (19.08593°N, 109.12275°E), from soil sample, 14 October 2023, Yi-Xin Wang (living culture SAUCC 6946-1).

##### GenBank accession numbers.

CGMCC 3.27033 (ITS, PP195857; LSU, PP195958; TEF, PP50103; ACT, PP933951; RPB1, PP883965), and SAUCC 6946-1 (ITS, PP1195858; LSU, PP195959; TEF, PP850104; ACT, PP933952; RPB1, PP883964).

##### Notes.

Based on phylogenetic analyses of ITS+LSU+TEF+ACT+RPB1 sequences, the two isolates of the new species *Gongronellabawanglingensis* form an independent clade with full support (MLBV = 100, BIPP = 1; Fig. 1), which is closely related to *G.inconstans* and *G.qichaensis*. In ITS, *G.bawanglingensis* differs from *G.inconstans* by 21 base pairs. This new species differs morphologically from *G.inconstans* in columella, apophysis, colour and sporangiospore. *G.bawanglingensis* and *G.inconstans* are similar in the dominant shape of columellae, but the former is longer than that of the latter (2.1–7.2 μm vs. 2.0–3.9 μm). As for apophyses, *G.bawanglingensis* and *G.inconstans* are remarkably different from each other in shape and size, the former mostly oval, 3.9–20.6 × 3.3–12.9 μm, sometimes subglobose, 4.8–12.2 × 4.7–12.3 μm, occasionally gourd-shaped, the latter mostly long fusiform, 7.6–17.4 × 5.4–4.7 μm, sometimes oval, 5.5–8.8 × 4.4–6.3 μm, rarely egg-shaped, 5.0–6.4 × 4.2–5.7 μm. As for collars, the *G.inconstans* are more distinct than *G.bawanglingensis* (2.0–17.0 μm vs. 0.7–5.9 μm). As for sporangiospores, *G.bawanglingensis* and *G.inconstans* are similar in dominant shape, but the former is smaller in size than the latter (ovoid, 2.5–3.6 × 1.7–2.6 μm vs. 2.7–4.9 × 1.8–3.5 μm, reniform, 2.6–3.3 × 1.9–2.2 μm vs. 3.1–4.1 × 2.0–4.5 μm). Additionally, the *G.inconstans* has more shapes, except ovoid and reniform. In ITS, *G.bawanglingensis* differs from *G.qichaensis* by 28 base pairs. This new species differs morphologically from *G.qichaensis* in sporangium, columellae and apophysis (Wang et al. 2023a). The *G.bawanglingensis* has evidently smaller sporangia than *G.qichaensis*, 4.2–18.5 μm vs. 7.9–36.7 μm. In columella and apophysis, the two species have evident differences in shape. Combining morphological and molecular phylogenetic analyses, we classified the two isolates together as a new species *G.bawanglingensis* allied to *G.inconstans* and *G.qichaensis*.

#### ﻿ Gongronella inconstans

Taxon classificationFungiMucoralesCunninghamellaceae

Yi Xin Wang, H. Zhao & X.Y. Liu sp. nov.

92DF5D15-CA6D-5A3F-B2E7-49765B7D306D

Fungal Names: FN 571905

Fig. 5

##### Etymology.

The epithet “*inconstans*” (Latin) refers to the inconstant shape of apophyses.

##### Type.

China • Hainan Province, Lingshui Li Autonomous County (18.69850°N, 109.88098°E), from soil sample, 7 Apr 72023, Yi-Xin Wang (holotype HMAS 352731, ex-holotype strain CGMCC 3.27029).

##### Description.

Colonies growing slowly on PDA in darkness at 25 °C, reaching 31.2–36.8 mm in diameter in seven days, white, regular at edge and cottony, reversely milky white. Rhizoids hyaline, branched, irregular, ubiquitous. Stolons absent. Sporangiophores on aerial mycelia, erect or slightly curved, unbranched or slightly branched (2–3 times), 1.7–3.9 μm width, hyaline, smooth, mostly aseptate. Fertile sporangia hyaline or light yellow, spherical, 8.8–21.4 μm in diameter, smooth and deliquescent-walled, leaving a collar after releasing sporangiospores. Columellae mostly hemispherical, 1.2–2.4 × 2.0–3.9 μm, sometimes spherical, 3.2–7.2 × 3.4–7.2 μm, hyaline, smooth. Collars distinct, 2.0–17.0 μm wide. Apophyses hyaline, smooth, variously shaped, mostly long fusiform, 7.6–17.4 × 4.7–5.4 μm, sometimes oval, 5.5–8.8 × 4.4–6.3 μm, rarely egg-shaped, 5.0–6.4 × 4.2–5.7 μm. Sporangiospores not uniform, hyaline, smooth, mostly ovoid, 2.7–4.9 × 1.8–3.5 μm, sometimes reniform, 3.1–4.1 × 2.0–4.5 μm or subglobose, 2.4–4.1 μm in diameter, occasionally irregular, 5.0–8.0 × 2.5–3.2 μm. Chlamydospores present, gourd-shaped and irregular. Giant cells intercalary, globular, 4.2–8.0 μm in diameter. Zygospores not found.

**Figure 5.**
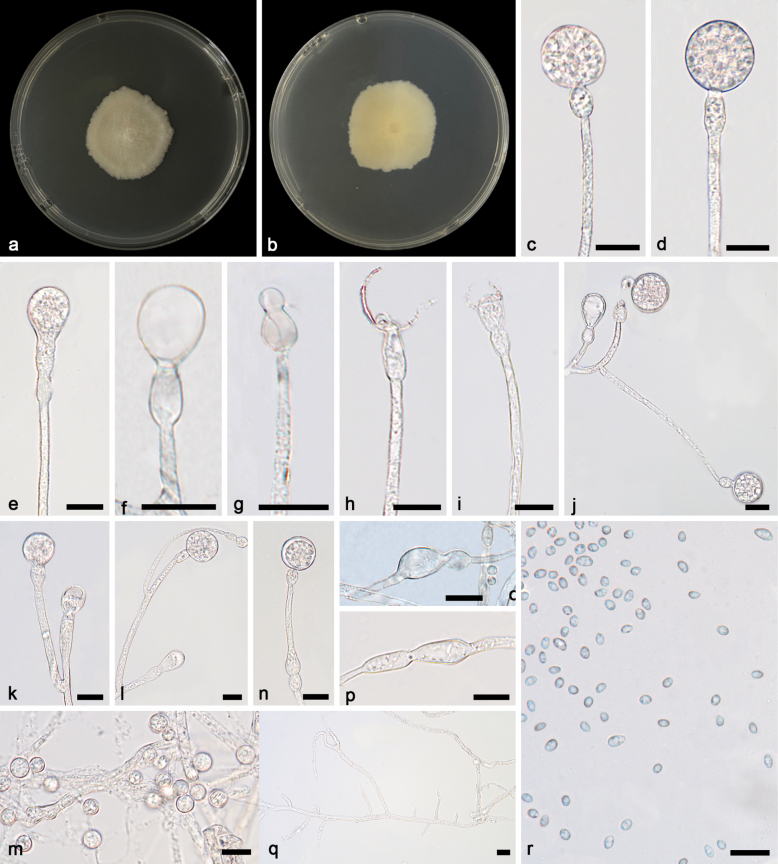
*Gongronellainconstans* ex-holotype CGMCC 3.27029 **a**, **b** colonies on PDA (**a** obverse **b** reverse) **c**, **d** an unbranched sporangiophore with a fertile sporangium **e** an unbranched sporangiophore with a premature sporangium **f** branched sporangiophores with aborted sporangia **g–i** columellae, collars, apophyses **j–l** branched sporangiophores with fertile sporangia, columellae, collars and apophyses **n** fertile sporangium with protuberance **o, p** chlamydospores **m** giant cells **q** rhizoids **r** sporangiospores. Scale bars: 10 μm (**c–r**).

##### Additional specimen examined.

China • Hainan Province, Lingshui Li Autonomous County (18.69850°N, 109.88098°E), from soil sample, 7 April 2023, Yi-Xin Wang (living culture SAUCC 4113-1).

##### GenBank accession numbers.

CGMCC 3.27029 (ITS, PP1955849; LSU, PP195950; TEF, PP850091; ACT, PP933941; RPB1, PP842874), and SAUCC 4113-1 (ITS, PP105850; LSU, PP195951; TEF, PP850092, ACT, PP933942; RPB1, PP842873).

##### Note.

Based on phylogenetic analyses of ITS+LSU+TEF+ACT+RPB1 sequences, the two isolates of the new species *Gongronellainconstans* form an independent clade with full support (MLBV = 100, BIPP = 1; Fig. 1), which is closely related to *G.qichaensis* with high support (BIPP = 0.96; Fig. 1). In ITS, *G.inconstans* differs from *G.qichaensis* by 28 base pairs. This new species differs morphologically from *G.inconstans* in sporangium, columellae and apophysis. As for sporangium, the *G.inconstans* is smaller than the *G.qichaensis*, 8.8–21.4 μm vs. 7.9–36.7 μm. The *G.inconstans* and *G.qichaensis* are different in size and shape of columellae (Wang et al. 2023a). The *G.inconstans* mostly hemispherical, 1.2–2.4 × 2.0–3.9 μm, sometimes spherical, 3.2–7.2 × 3.4–7.2 μm. Additionally, the columellae of *G.qichaensis* is mostly ellipsoidal, 0.8–6.5 × 1.2–8.1 µm, sometimes sub-hemispherical to curved, 1.0–2.0 × 2.5–4.5 µm. *G.inconstans* and *G.qichaensis* are evidently different in apophysis shape. The former mostly long fusiform, sometimes oval-shaped and rarely egg shaped. The latter mostly pear-shaped to oval, partially elliptical or sub-globose. Combining morphological and molecular phylogenetic analyses, we classified the two isolates together as a new species: *G.inconstans* allied to *G.qichaensis*.

#### ﻿ Gongronella pamphilae

Taxon classificationFungiMucoralesCunninghamellaceae

Y.P. Tan, Bishop-Hurley & R.G. Shivas

B30FB7F4-63FA-5B58-88DD-1A927935385D

Fungal Names: FN 900776

Fig. 6

##### Etymology.

Named after Pamphilae of Epidaurus (ca. 1^st^ century AD), a historian of Egyptian descent who lived in Greece.

##### Description.

Colonies growing slowly on PDA in darkness at 25 °C, reaching 36.6–44.6 mm in diameter in seven days, white, regular at edge and cottony in the centre, reversely milky white. Rhizoids hyaline, branched, irregular. Stolons absent. Sporangiophores on aerial mycelia, erect or slightly curved, unbranched or slightly branched (1–2 times), 3.7–154.9 × 1.4–4.1 μm, hyaline, smooth, mostly aseptate, no more than two-septate. Fertile sporangia hyaline or light yellow, spherical, 13.8–30.8 μm in diameter, smooth and deliquescent-walled, leaving a collar after releasing sporangiospores. Columellae mostly hemispherical, 1.8–4.7 × 2.0–7.7 μm, sometimes arc-shaped, 0.5–1.6 × 3.3–4.6 μm, occasionally subglobose, 4.8–6.4 × 5.9–6.9 μm, hyaline, smooth. Collars distinct, 1.0–5.1 μm wide. Apophyses hyaline, smooth, variously shaped, mostly subglobose, 5.7–8.1 × 5.6–9.0 μm, sometimes ellipsoidal, 4.8–6.9 × 4.8–6.1 μm. Sporangiospores not uniform, hyaline, smooth, reniform, 3.0–5.5 × 1.8–3.4 μm, ovoid, 2.5–5.6 × 1.8–3.7 μm. Chlamydospores present, ellipsoidal. Giant cells intercalary, globose, 4.0–8.1 μm in diameter. Zygospores not found.

**Figure 6.**
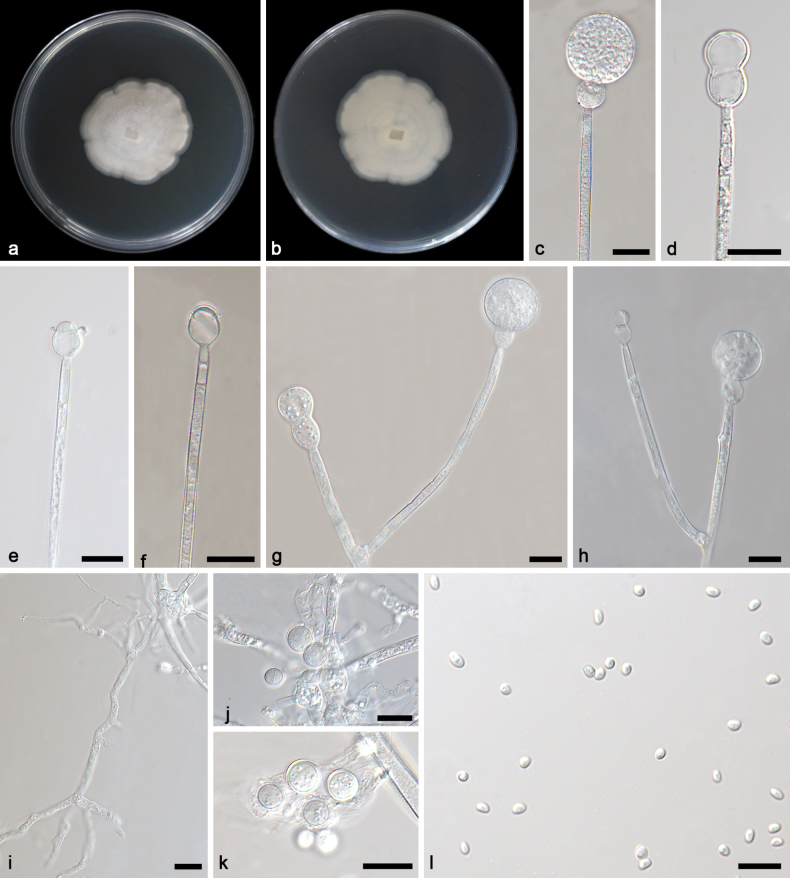
*Gongronellapamphilae* ex-living culture CGMCC 3.27027 **a**, **b** colonies on PDA (**a** obverse **b** reverse) **c** an unbranched sporangiophore with a fertile sporangium **d** an unbranched sporangiophore with an aborted sporangium **e, f** an unbranched sporangiophore with columellae, apophyses and collars **g, h** branched sporangiophores with columellae, collars, apophyses **i** Rhizoids **j, k** giant cells **l** sporangiospores. Scale bars: 10 μm (**c–l**).

##### Additional specimen examined.

China • Hainan Province, Lingshui Li Autonomous County, Shizhi Village Road (18.70178°N, 109.83679°E), from soil sample, 10 April 2023, Yi-Xin Wang (specimen HMAS 352732, living culture CGMCC 3.27027, SAUCC 4031-2).

##### GenBank accession numbers.

CGMCC 3.27027 (ITS, PP195845; LSU, PP195946; TEF, PP850086; ACT, PP933935; RPB1, PP850081), and SAUCC 4031-2 (ITS, PP195846; LSU, PP195947; TEF, PP850085; ACT, PP933936; RPB1, PP850082).

##### Note.

Based on phylogenetic analyses of ITS+LSU+TEF+ACT+RPB1 DNA sequences, the two isolates of the new record species *Gongronellapamphilae* form an independent clade with full support (MLBV = 100; Fig. 1), which is closely related to *G.pamphilae* (MLBV = 100; BI = 1, Fig. 1). In ITS, the two isolates differ from *G.pamphilae* by only 2 base pairs. As no morphological descriptions were provided for the *G.pamphilae* in its protologue, we classified the two isolates together as members of *G.pamphilae* just based on molecular phylogenetic analyses. Consequently, we provide herein a supplemental description for the species.

#### ﻿ Gongronella pingtangensis

Taxon classificationFungiMucoralesCunninghamellaceae

Yi Xin Wang, H. Zhao & X.Y. Liu sp. nov.

2BEBB187-42DE-5630-A2D9-2BEA6C838CA6

Fungal Names: FN 571904

Fig. 7

##### Etymology.

The epithet “*pingtangensis*” (Latin) refers to the location where the type was collected, Pingtang County.

##### Type.

China • Qiannan Buyi and Miao Autonomous Prefecture, Pingtang County, Kapu Maonan Town (25.79510°N, 107.38631°E), from soil sample, 7 August 7 2023, Yi-Xin Wang (holotype HMAS 352732, ex-holotype strain CGMCC 3.27032).

##### Description.

Colonies growing slowly on PDA in darkness at 25 °C, reaching 38.8–45.6 mm in diameter in seven days, white, cottony, in reverse milky white. Rhizoids hyaline, branched, irregular. Stolons absent. Sporangiophores on aerial mycelia, erect or slightly curved, unbranched, or slightly branched (1–4 times), sympodially branched, 1.4–5.9 μm in width, hyaline, smooth, mostly aseptate or one-septate. Sterile (aborted) sporangia predominantly on the top of short lateral branches of sporangiophores. Fertile sporangia hyaline or light yellow, spherical, 14.2–27.1 μm in diameter, smooth and deliquescent-walled, leaving a collar after releasing sporangiospores. Columellae mostly hemispherical, 2.3–4.0 × 2.8–6.9 μm, partially arch-shaped, 0.9–1.5 × 4.1–4.9 μm, rarely spherical, 4.4–6.0 × 5.1–6.9 μm, hyaline, smooth. Collars mostly distinct, 0.6–8.7 μm wide. Apophyses hyaline, smooth, variously shaped, mostly oval, 7.1–19.8 × 6.9–15.9 μm, partially bowling pin-shaped, 15.6–17.5 × 8.5–9.4 μm, rarely egg-shaped, 4.6–9.8 × 3.6–8.7 μm. Sporangiospores not uniform, hyaline, smooth, mostly ovoid, 2.8–3.9 × 2.0–2.5 μm, sometimes reniform, 2.9–3.6 × 1.9–2.4 μm and globose, 2.1–2.7 μm in diameter, occasionally irregular, 4.8–6.2 × 2.1–2.8 μm. Chlamydospores absent. Giant cells in rhizoids, intercalary, globose, 5.2–6.8 μm in diameter. Zygospores not found.

**Figure 7.**
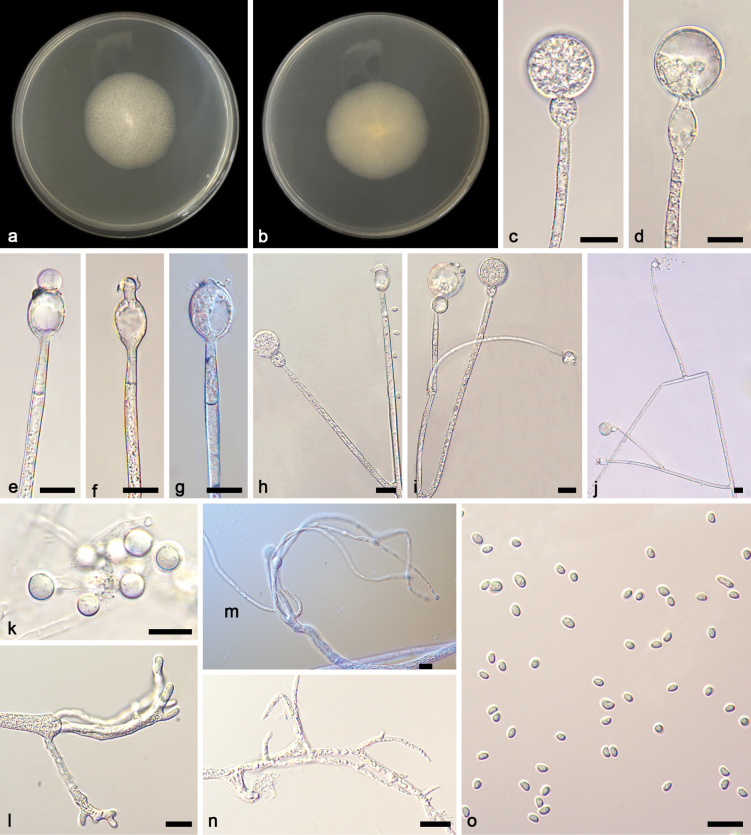
*Gongronellapingtangensis* ex-holotype CGMCC 3.27032 **a**, **b** colonies on PDA (**a** obverse **b** reverse) **c**, **d** an unbranched sporangiophore with a fertile sporangium **e–g** columellae, collars, apophyses and septa **h–j** branched sporangiophores with fertile sporangia, columellae, collars, apophyses and septa **k** giant cells **i–n** rhizoids **o** sporangiospores. Scale bars: 10 μm (**c–o**).

##### Additional specimen examined.

China • Qiannan Buyi and Miao Autonomous Prefecture, Pingtang County, Kapu Maonan Town (25.79510°N, 107.38631°E), from soil sample, 7 August 2023, Yi-Xin Wang (living culture SAUCC 5676-2).

##### GenBank accession numbers.

CGMCC 3.27032 (ITS, PP195855; LSU, PP195956; TEF, PP850101; ACT, PP933949; RPB1, PP842880), and SAUCC 5676-4 (ITS, PP195856; LSU, PP195957; TEF, PP850102; ACT, PP933950; RPB1, PP842879).

##### Note.

Based on phylogenetic analyses of ITS+LSU+TEF+ACT+RPB1 sequences, the two isolates of the new species *G.pingtangensis* form an independent clade with high support (MLBV = 100, BIPP = 0.84; Fig. 1), which is closely related to *G.namwonensis* with high support (BIPP = 1; Fig. 1). In ITS, *G.pingtangensis* differs from *G.namwonensis* by 14 base pairs. This new species differs morphologically from *G.namwonensis* in columellae, apophysis and giant cell (Crous et al. 2020). *G.pingtangensis* and *G.namwonensis* greatly differ from each other in shape of columellae, the former being mostly hemispherical, partially arch-shaped, rarely spherical and the latter being globose, subglobose, hemispherical, nipple-like and ellipsoidal. As for apophyses, *G.pingtangensis* and *G.namwonensis* obviously differ from each other in shape, the former being mostly oval, partially bowling pin-shaped, rarely egg-shape and the latter being subglobose and ellipsoid, sometimes with a truncated base. As for giant cells, the *G.namwonensis* varies in shape more than *G.pingtangensis*. Combining morphological and molecular phylogenetic analyses, we classified the two isolates together as a new species *G.pingtangensis* allied to *G.namwonensis*.

#### ﻿ Gongronella reniformis

Taxon classificationFungiMucoralesCunninghamellaceae

Yi Xin Wang, H. Zhao & X.Y. Liu sp. nov.

02DD5FD9-57CE-5086-9663-46C81352A6F0

Fungal Names: FN 571630

Fig. 8

##### Etymology.

The epithet “*reniformis* “ (Latin) refers to the reniform sporangiospores.

##### Type.

China • Hainan Province, Changjiang Li Autonomous County, Qicha Town (19.11750°N, 109.15000°E), from soil sample, 11 April 2023, Yi-Xin Wang (holotype HMAS 352727, ex-holotype strain CGMCC 3.27030).

##### Description.

Colonies on PDA in darkness at 25 °C growing slowly, reaching 39.4–41.8 mm in diameter in seven days, white, regular at edge and cottony in the centre, on reverse milky white. Rhizoids hyaline, branched, irregular, sometimes with giant cells in the terminal. Stolons absent. Sporangiophores on aerial mycelia, erect or slightly curved, unbranched or slightly branched (1–3 times), 3.4–157.9 × 0.8–3.4 μm, hyaline, smooth, mostly aseptate, partially no more than two-septate. Sterile (aborted) sporangia predominantly on the top of short lateral branches of sporangiophores, gourd-shaped, 15.0–19.9 × 3.1–10.9 μm. Fertile sporangia hyaline or light yellow, spherical, 7.9–26.0 μm in diameter, smooth and deliquescent-walled, leaving a collar after releasing sporangiospores. Columellae mostly elliptic, 1.7–4.6 × 1.4–5.2 μm, sometimes sub-hemispherical, 1.4–2.6 × 3.3–4.9 μm, hyaline, smooth. Collars distinct, 2.1–4.3 μm. Apophyses hyaline, smooth, variously shaped, pear-shaped, 3.3–8.5 × 3.0–7.3 μm, ellipsoidal, 4.6–10.1 × 2.9–7.8 μm. Sporangiospores not uniform, hyaline, smooth, mostly reniform, 2.8–3.5 × 1.8–2.3 μm, occasionally ovoid, 3.1–3.4 × 1.7–2.0 μm. Chlamydospores, mostly ellipsoidal, 7.3–12.5 × 6.1–11.2 μm, sometimes irregular. Giant cells intercalary, globose, 3.5–10.0 μm in diameter. Zygospores not found.

**Figure 8.**
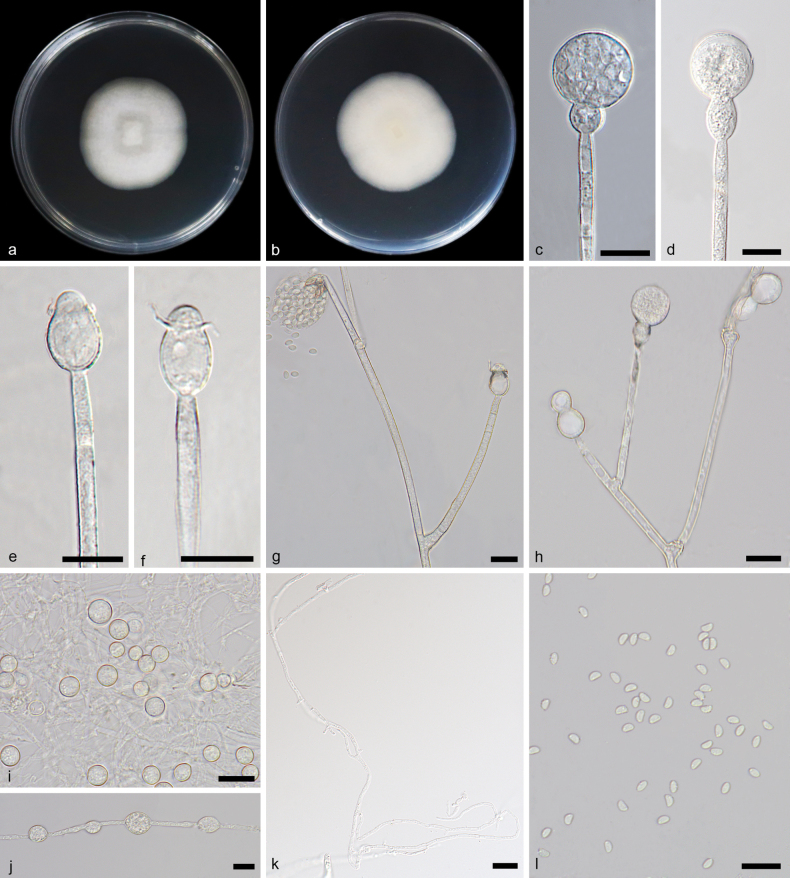
*Gongronellareniformis* ex-holotype CGMCC 3.27030 **a**, **b** colonies on PDA (**a** obverse **b** reverse) **c** an unbranched sporangiophore with a fertile sporangium **d** an unbranched sporangiophore with an immature sporangium **e**, **f** columellae, collars and apophyses **g** branched sporangiophores with shedding sporangia, columellae, collars, apophyses and septa **h** branched sporangiophores with fertile sporangia and sterile (aborted) sporangia **i** giant cells **j** chlamydospore **k** rhizoids **l** sporangiospores. Scale bars: 10 μm (**c–l**).

##### Additional specimen examined.

China • Hainan Province, Changjiang Li Autonomous County, Qicha Town (19.11750°N, 109.15000°E), from soil sample, 11 April 2023, Yi-Xin Wang (living culture SAUCC 4142-5).

##### GenBank accession numbers.

CGMCC 3.27030 (ITS, PP195851; LSU, PP195952; TEF, PP850095; ACT, PP933943; RPB1, PP84875), SAUCC 4142-5 (ITS, PP195852; LSU, PP195953; TEF, PP850096; ACT, PP933944; RPB1, PP842876).

##### Notes.

Based on phylogenetic analyses of ITS+LSU+TEF+ACT+RPB1 sequences, the two isolates of the new species *Gongronellareniformis* form an independent clade with full support (MLBV = 100, BIPP = 1; Fig. 1), which is close to *G.pamphilae* and *G.brasiliensis* with a high support (MLBV = 89, BIPP = 1; Fig. 1). Comparing ITS sequences showed that *G.reniformis* is relatively closely related to *G.pamphilae* (44 bp of dissimilarity) and *G.brasiliensis* (40 bp of dissimilarity). There were no morphological descriptions of *G.pamphilae* in its protologue, so the morphological comparison was made between *G.reniformis* and the *G.pamphilae* strains identified in this study. This new species differs morphologically from *G.pamphilae* in sporangium, columellae, apophysis, sporangiospore. The sporangium of *G.reniformis* is smaller than that of *G.pamphilae* (7.9–26.0 μm vs. 13.8–30.8 μm). *G.reniformis* and *G.pamphilae* are different from each other mainly in shape and size of columellae, the former being mostly elliptic, 1.7–4.6 × 1.4–5.2 μm, sometimes sub-hemispherical, 1.4–2.6 × 3.3–4.9 μm and the latter being mostly hemispherical, 1.8–4.7 × 2.0–7.7 μm, sometimes arc-shaped, 0.5–1.6 × 3.3–4.6 μm. The *G.reniformis* and *G.pamphilae* are different from each other in dominant shape and size of apophyses, the former being pear-shaped, 3.3–8.5 × 3.0–7.3 μm and ellipsoidal, 4.6–10.1 × 2.9–7.8 μm, the latter being spherical, 5.7–8.1 × 5.6–9.0 μm and ellipsoidal, 4.8–6.9 × 4.8–6.1 μm. The sporangiospores of *G.reniformis* are smaller than those of *G.pamphilae* (reniform, 2.8–3.5 × 1.8–2.3 μm vs. 3.0–5.5 × 1.8–3.4 μm, ovoid, 3.1–3.4 × 1.7–2.0 μm vs. 2.5–5.6 × 1.8–3.7 μm). This new species differs morphologically from *G.brasiliensis* in sporangiophore, columellae and giant cells (Tibpromma et al. 2017). In sporangiophores, the *G.renformis* differs from the *G.brasiliensis* in size, 3.4–157.9 × 0.8–3.4 μm vs. 26.5–320.0 × 2.5–5.0 μm. As for columellae, the *G.renformis* and *G.brasiliensis* are different in shape. The former mostly elliptic, sometimes sub-hemispherical. The latter globose, subglobose and conical-cylindrical. The *G.renformis* is evidently smaller than *G.brasiliensis* in giant cells, 3.5–10.0 μm vs. up to 48 μm. Combining morphological and molecular phylogenetic analyses, we classified the two isolates as a new species *G.reniformis*.

### ﻿Morphological comparisons and key to the species of *Gongronella*

Together with the six new species proposed in this study, a total of 25 species of *Gongronella* have been described worldwide. Except *G.banzhaoae*, morphological comparisons were made amongst 18 species published before and six species newly proposed in this study (Table 4). We provide herein a synoptic key for these species. Characteristics adopted in the key include colonies, sporangiophores, sporangia, columellae, apophyses, sporangiospores and giant cells.

**Table 4.** Morphological comparisons of *Gongronella* species.

Species	Colonies	Sporangiophores	Sporangia	Columellae	Apophyses	Sporangiospores	Giant cell	Reference
*G.abortosporangia*	PDA: dark 25 °C 7 d 24.6–26.2 mm diam., white, regular at edge and cottony in the centre, in reverse milky white	unbranched or branched 1–6 times, 4.0–96.8 × 1.0–4.2 μm, mostly aseptate, partially 1-septate, rarely 2-septate, occasionally containing a line of oil droplets	Aborted: mostly gourd-shape, 11.6–16.7 × 5.5–17.7 μm, partially elliptical with slight shrinkage, 12.5–18.0 × 6.7–10.6, occasionally clavate, 20.1–22.7 × 9.5–10.4 μm; Fertile: 7.0–23.2 μm diam	mostly hemispherical, 2.5–4.2 × 3.6–7.4 μm, sometimes sub-hemispherical, 1.3–3.9 × 3.6–5.5 μm	mostly cup-shaped, 1.9–8.6 × 2.1–6.7 μm, partially hemispherical, 2.7–5.5 × 2.8–7.4 μm, occasionally pear-shaped, 8.2 × 7.2 μm	ovoid, 2.6–3.5 × 1.7–2.1 μm, reniform, 2.9–3.5 × 1.7–2.3 μm	intercalary, globular, subglobular, 2.6–4.6 μm diam.	This study
*G.apophysata*	PDA: dark 25 °C 7 d 17.9–21.2 mm in diam., white, irregular at edge and cottony in centre, in reverse milky white	unbranched or branched 1–2 times, 11.2–190.9 × 1.6–3.9 μm, mostly aseptate or 1-septate, occasionally 2-septate	Aborted: gourd-shape, 14.0 × 8.3 μm; Fertile: spherical, 12.5–40.5 μm diam.	elliptic, 2.6–4.0 × 2.1–5.5 μm, sub-hemispherical, 1.4–2.7 × 2.2–4.3 μm	mostly ellipsoidal to olivary, 2.3–17.3 × 2.4–10.0 μm, partially subglobose, 4.6–10.2 × 4.3–10.0 μm, occasionally gourd-shaped, 11.4 × 4.9 μm	mostly reniform, 3.2–5.5 × 1.7–3.1 μm, ovoid, 2.5–3.7 × 1.7–2.6 μm, occasionally sub-orbicular, 1.7–2.5 μm	intercalary, globular, 4.4–10.5 μm diam.	This study
G.bawanglingensis	PDA: dark 25 °C 7 d 22.8–24.4 mm diam., white, cottony in centre, in reverse milky white	unbranched or sympodially branched 1–3 times, 1.3–4.5 μm wide, mostly aseptate or 1-septate, occasionally up to 4-septate	Aborted: mostly gourd-shaped; Fertile: spherical, 4.2–18.5 μm diam.	mostly hemispherical, 1.6–5.1 × 2.1–7.2 μm, some arch-shaped, 1.4–3.7 × 2.6–8.8 μm, spherical, 2.3–6.1 × 2.5–8.1 μm	oval-shaped, 3.9–20.6 × 3.3–12.9 μm, subglobose-shaped, 4.8–12.2 × 4.7–12.3 μm, occasionally gourd-shaped	mostly ovoid, 2.5–3.6 × 1.7–2.6 μm, reniform, 2.6–3.3 × 1.9–2.2 μm	intercalary, globular, 3.2–6.9 μm diam.	This study
*G.brasiliensis*	MEA: 25 °C 7 d 1.0–2.0 mm high 60.0 mm diam., white, cottony, irregular at edge, reverse cream to buff	26.5–320.0 × 2.5–5.0 µm, solitary, arising from stolons or in whorls of two, often with a single branch, 1- or 2-septate below apophyses	Aborted: globose, 5.0–17.0 µm diam.; Fertile: globose, subglobose, 9.5–30.0 μm diam.	globose, subglobose, (3.0–)4.0–8.0(–9.0) μm, conical-cylindrical, 1.5–2.5 × 2.0–3.0 μm, some very small, up to 1 μm diam.	globose, (3.0–)4.0–5.0(–6.0) μm diam., vase-shaped, (3.0–)4.0 × 12.0(–14.5) μm, ellipsoidal, 5.0–10.0(–12.0) × 3.0–7.0(–8.5) μm	reniform, 1.5–4.0 × 1.5–2.5 μm, ellipsoid to fusiform, 2.0–6.5 × 1.5–3.0 μm, ellipsoid with a flattened end, 2.5–7.5 × 1.5–4.0 μm	globose, subglobose, ovoid, some hypha-like, irregularly swollen, up to 48.0 μm diam.	Tibpromma et al. (2017)
*G.butleri*	White turf	simply or irregularly branched, 2.1–3.1 μm wide, always 1-septate	Fertile: globose, 16.5–22.7 μm		swollen, oval-shaped, 7.0–10 × 8.0–8.7 μm	oval to flattened on one side to reniform, 2.5–7.2 × 1.7–4.7 μm		Ribaldi (1952), Babu et al. (2015)
*G.chlamydospora*	PDA: 27 °C 11 d 90.0 mm diam., floccose, at first white, then drab grey	unbranched or simply branched, hyaline, slightly constricted at top	Fertile: globose, 8.5–17.0 μm diam.	ovoid to depressed subglobose, 3.0–5.5 × 3.5–6.5 μm	urn-shaped to subglobose, 6.0–12.0 × 6.0–10.0 μm	ellipsoid, reniform or irregular, 2.0–3.0 × 1.0–2.0 μm		Zhao et al. (2023)
*G.eborensis*	PDA: 25 °C 5 d 28.0–32.0 mm diam.	46.0–94.0 × 1.5–3.0 µm, irregularly or simply branched, always 1-septate under apophyses	Fertile: globose to subglobose, 7.5–16.0 × 7.0–13.0 µm	hemispherical to subglobose, 11.5–5.5 × 8.2–3.2 µm	globose to subglobose, 3.5–6.5 × 3.0–7.0 µm	reniform to fusiform-elliptical, 2.6–3.8 × 1.2–1.6 µm		Martins et al. (2020)
*G.guangdongensis*	PDA: 25 °C 13 d 1–2 mm high, 50.0 mm diam., white or pale, irregular at edge; in reverse buff to honey	irregularly or simply branched, 28.0–100.0 × 2.0–2.5 μm, always 1-septate	Aborted: sometimes present; Fertile: always globose, 14.0–21.5 μm diam.	hemispherical, spherical or ovoid, 2.5–12.0 × 2.0–12.0 μm	hemispherical, 5.5–9.0 μm in diam.	globose, 2.0–3.0 μm in diam.		Adamcik et al. (2015)
*G.hydei*	PDA: 25 °C 7 d 60.0–65.0 mm diam., circular, entire at edge, flat or effuse, dense, white;	up to 120.0 µm long, 1.6–3.2 µm wide, mostly unbranched, occasionally branched, mostly 1-septate	Fertile: globose to subglobose, 10.5–18.8 × 10.0–17.5 µm	hemispherical, sometimes tiny, 1.7–4.7 × 2.2–6.3 µm	cuboid-shaped with truncate at the base, 2.5–3.9 × 3.5–5.1 µm; cup-shaped rounded at the base, 2.7–6.2 × 3.8–7.8 µm; cup-shaped truncate at the base, 3.7–7.3 × 3.8–7.3 µm	reniform, 2.4–3.8 × 1.5–2.3 µm, ellipsoidal to fusiform, 2.6–3.4 × 1.8–3.4 µm	globose, guttulate, up to 25.0 µm diam.	Doilom et al. (2020)
*G.inconstans*	PDA: dark 25 °C 7 d 15.6–18.4 mm diam., white, regular at edge, cottony, in reverse milky white	unbranched or branched 2–3 times, 1.7–3.9 μm wide, mostly aseptate	Aborted: existence; Fertile: spherical, 8.8–21.4 μm diam.	mostly hemispherical, 1.2–2.4 × 2.0–3.9 μm, sometimes spherical, 3.2–7.2 × 3.4–7.2 μm	variously shaped, mostly long fusiform, 7.6–17.4 × 4.7–5.4 μm, sometimes oval-shaped, 5.5–8.8 × 4.4–6.3 μm, rarely egg-shaped, 5.0–6.4 × 4.2–5.7 μm	ovoid, 2.7–4.9 × 1.8–3.5 μm, reniform, 3.1–4.1 × 2.0–4.5 μm, some subcircular, 2.4–4.1 μm, occasionally irregular, 5.0–8.0 × 2.5–3.2 μm	intercalary, globular, 4.2–8.0 μm diam.	This study
*G.koreana*	PDA: 25 °C 7 d 31.5–33.0 mm diam., light white at first, cotton white with age, reverse from light-coloured to white	2.5–2.8 μm wide, mostly branched, 1-septate	Fertile: globose, 12.3–15.5 × 12.4–15.6 μm diam.	hemispherical, 1.2–2.3 × 2.6–3.3 μm	typically pyriform, 5.4–6.5 × 5.9–7.1 μm	mostly bean-shaped, 1.7–2.1 × 2.1–2.8 μm		Ariyawansa et al. (2015)
*G.lacrispora*	25 °C 13 d 50.0 mm in diam., 1–3 mm high, thickly floccose to felty, irregular at edge, at first white, then grey or pale grey, later pale wine colour	up to 6.5 μm wide, rarely septate	Aborted: sometimes present; Fertile: typically perfectly globose, 13.0–41.0 μm diam	dorsiventrally flattened to spherical, 2.5–13.0 × 4.5–20.0 μm	hemispherical, 4.0–8.6 μm. in diam.	lacrymoid to narrowly napiform, 2.8-4.5 × 5.5-9.0 μm	intercalary, globose to irregular, often with vacuoles or oil droplets, 20.0-37.0 × 60.0 μm	Hesseltine and Ellis (1961)
*G.multiramosa*	PDA: dark 25 °C 7 d 21.6–25.6 mm diam., white, regular at edge, cottony in centre, reverse milky white	unbranched or sympodially branched up to 7 times, 4.7–128.4 × 2.6–3.9 µm, usually 1-septate, occasionally containing a line of oil droplets	Aborted: ovoid, 9.6 × 6.2 µm diam.; Fertile: spherical, 15.5–23.2 µm diam.;	mostly hemispherical, 3.6–5.7 × 8.0–9.8 µm, sometimes sub-hemispherical, 3.0; –3.9 × 7.6–10.0 µm	mostly hemispherical, 4.4–5.6 × 8.5–9.0 µm, partially cup-shaped,4.6–7.0 × 8.5–10.0 µm	subspherical, 1.7–2.6 µm, ovoid, 2.6–3.3 × 1.7–2.3 µm, few reniform, 2.7–3.4 × 1.3–1.9 µm	globular, sub-spherical, 3.0–6.7 µm diam.	Wang et al. (2023a)
*G.multispora*	PDA: 27 °C 10 d 80.0 mm diam., 10.0 mm high, from white to yellowish, in reverse crusty, yellow	unbranched or sympodially branched, 2–3 in whorls and swollen on the base, 1 to several septate	Fertile: globose, 12.0–17.0 μm diam.	hemispherical, 2.0–4.5 × 2.0–4.0 μm	pyriform to subglobose, 8.0–12.0 × 7.0–9.5 μm	ellipsoid, fusiform, cylindrical, reniform subglobose to globose or irregular, 2.5–3.5 × 1.5–2.5 μm		Zhao et al. (2023)
*G.namwonensis*	MEA:25 °C 7 d 55.0 mm diam. (28 °C 5 d 90.0 mm diam), white, in reverse cream	simply or sympodially or monopodially branched, up to 1 mm long and 5.0 μm wide, in whorls of 2 or 3 times, mostly 1-septate	Aborted: sometimes formed; Fertile: globose, up to 30.0 µm diam.	globose, subglobose, 3.5–7.0 µm diam., hemispherical,; 1.8–5.5 × 2.5–8.5 µm, nipple-like, ellipsoidal, 2.0–3.8 × 2.0–5.0 µm	globose (2.5–)5.0–9.5(–12.0); µm, subglobose and ellipsoid, some with a truncated base,; 7.5–14.5 × 5.5–12.0 µm	reniform, ellipsoidal, some ovoid, 2.5–3.5 × 1.7–2.5 µm, rarely irregular, up to 6 × 2.5 µm	globose, subglobose and branched	Crous et al. (2020)
*G.oleae*	PDA: dark 25 °C 7 d, 16.3–17.0 mm diam., white, regular at edge, cottony in centre, inreverse milky white	unbranched or branched 3–4 times, 7.0–96.8 × 0.9–3.5 µm, mostly aseptate, sometimes 1-septate	Aborted: 7.0–7.8 µm diam.; Fertile: spherical, 8.8–24.5 µm diam.;	mostly sub-spherical or ovoid, 2.6–5.2 × 3.2–6.5 µm, sometimes hemi-spherical, 0.4–3.3 × 2.8–5.3 µm	pear-shaped, 4.4–5.6 × 8.5–9.0 µm, cup-shaped, 4.6–7.0 × 8.5–10.0 µm, elliptical or subspherical, 2.7–8.0 × 2.8–9.1 µm	ovoid, 2.40–3.34 × 1.51–2.35 µm, reniform, 2.58–4.99 × 1.48–2.24 µm	terminal, globular, sub-spherical, 3.2–6.5 µm diam.	Wang et al. (2023a)
*G.orasabula*	SMA: 25 °C 5 d, 33.0–35.0 mm, initial white, later off-white, irregular at edge, in reverse white	35.0–200.0 × 2.5–4.0 μm, simply branched 1–3 times	Fertile: globose to subglobose or calabash vase-shaped, 12.0–20.0 × 12.5–22.0 μm	hemispherical, 2.0–3.0 × 3.0–4.0 μm	globose, subglobose to pyriform, 5.0–10.0 × 4.5–8.5 μm	mostly bean-shaped, 2.0–3.5 × 2.0–2.5 μm		Li et al. (2016)
*G.pamphilae*	PDA: dark 25 °C 7 d 18.3–22.3 mm in diam., white, regular at edge and cottony in centre, in reverse milky white	unbranched or branched 1–2 times, 3.7–154.9 × 1.4–4.1 μm, mostly aseptate, occasionally 1- or 2-septate	Aborted: existence; Fertile: spherical, 13.8–30.8 μm diam.	mostly hemispherical, 1.8–4.7 × 2.0–7.7 μm, sometimes arc-shaped, 0.5–1.6 × 3.3–4.6 μm, spherical, 4.8–6.4 × 5.9–6.9 μm	spherical, 5.7–8.1 × 5.6–9.0 μm, ellipsoidal, 4.8–6.9 × 4.8–6.1 μm	reniform, 3.0–5.5 × 1.8–3.4 μm, ovoid, 2.5–5.6 × 1.8–3.7 μm	intercalary, globular, 4.0–8.1 μm diam	This study
*G.pedratalhadensis*	PDA: 25 °C 7 d 5.5 mm high, 45.0 mm diam., white, irregular at edge, in reverse pale	sympodially branched 1–2 times, 9.5–30.0 × 2.5–7.0 μm, mostly 1-septate below sporangia, rarely two or more septate	Aborted: existence; Fertile: globose 17.0–35.0(40.0) μm diam.	mostly hemispherical, some short hemispherical or subglobose, 5.0–15.0 × 4.0–21.5 μm	vasiform, short or long, 5.0–15.0 × 4.5–15.0 μm	bean-shaped, 2.5–3.5 × 1.5–2.5 μm, rarely irregular, 2.5–3.5 × 2.0–3.0 μm		de Freitas et al. (2020)
*G.pingtangensis*	PDA: dark 25 °C 7 d 19.4–22.8 mm diam., white, cottony, in reverse milky white	unbranched or sympodially branched 1–4 times, 1.4–5.9 μm wide, aseptate or 1-septate	Aborted: existence; Fertile: spherical, 14.2–27.1 μm diam.;	mostly hemispherical, 2.3–4.0 × 2.8–6.9 μm, some arch-shaped, 0.9–1.5 × 4.1–4.9 μm, spherical, 4.4–6.0 × 5.1–6.9 μm	mostly oval-shaped, 7.1–19.8 × 6.9–15.9 μm, some bowling pin-shaped, 15.6–17.5 × 8.5–9.4 μm, egg-shaped, 4.6–9.8 × 3.6–8.7 μm	mostly ovoid, 2.8–3.9 × 2.0–2.5 μm, some reniform, 2.9–3.6 × 1.9–2.4 μm, spherical, 2.1–2.7 μm, occasionally large irregularly shaped, 4.8–6.2 × 2.1–2.8 μm	intercalary, globular, 5.2–6.8 μm diam.	This study
*G.qichaensis*	PDA: dark, 25 °C 7 d 20.3–22.7 mm diam., white, cottony, regular at edge, in reverse milky white	unbranched or branched 1–2 times, 17.3–141.2 × 0.7–4.3 µm, usually aseptate, occasionally 2-septate	Aborted: ovoid,12.2–13.7 µm in diam.; Fertile: spherical, 7.9–36.7 µm diam.	ellipsoidal, 0.8–6.5 × 1.2–8.1 µm, sometimes sub-hemispherical to curved, 1.0–2.0 × 2.5–4.5 µm	mostly pear-shaped to oval, 4.6–13.4 × 3.4–10.7 µm, partially elliptical or sub-spherical, 6.0–11.3 × 4.8–9.0 µm	mostly ellipsoidal, 3.0–4.2 × 2.1–2.8 µm, sometimes reniform, 2.8–3.7 × 2.3–2.8 µm, few spherical, 2.4–3.3 µm	intercalary or terminal, globular, sub-spherical, 3.5–6.7 µm diam.	Wang et al. (2023a)
*G.reniformis*	PDA: dark 25 °C 7 d 19.7–20.9 mm diam., white, regular at edge and cottony in centre in reverse milky white	unbranched or branched 1–3 times, 3.4–157.9 × 0.8–3.4 μm, mostly aseptate, occasionally 1- or 2-septate	Aborted: gourd-shape, 15.0–19.9 × 3.1–10.9 μm; Fertile: spherical, 7.9–26.0 μm diam.	mostly elliptic, 1.7–4.6 × 1.4–5.2 μm, sometimes sub-hemispherical, 1.4–2.6 × 3.3–4.9 μm	pear-shaped, 3.3–8.5 × 3.0–7.3 μm, ellipsoidal, 4.6–10.1 × 2.9–7.8 μm	mostly reniform, 2.8–3.5 × 1.8–2.3 μm, ovoid, 3.1–3.37 × 1.7–2.0 μm	intercalary, globular, 3.5–10.0 μm diam.	This study
*G.sichuanensis*	PDA: 25 °C 14 d 4.0–5.0 mm high, 67.0–68.0 mm diam., white, regular at edge, in reverse grey	solitary or simply branched, 28.0–46.5 × 1.0–3.0 μm, 1- or 2-septate	Fertile: globose, subglobose, 10.5–26.5 μm diam.	hemispherical, 1.5–3.5 × 1.0–3.0 μm	ellipsoidal to subglobose, 4.5–8.5 × 4.5–6.0 μm in diam.	reniform, ovoid or ellipsoidal, 1.5–2.0 × 1.0–1.5 μm		Zhang et al. (2019)
*G.zunyiensis*	PDA: 25 °C 14 d 3.0–6.0 mm high, 70.0–75.0 mm diam., white, villiform, irregular at edge, in reverse grey-white	1.5–4.0 μm wide, branched several times, usually aseptate	Fertile: subglobose to globose, 11.0–19.5 μm diam.	hemispherical and globose, 2.0–3.0 × 3.5–7.0 μm	subglobose, 3.5–9.5 μm, conical-cylindrical, 4.0–7.0 × 5.0–9.0 μm	subglobose, reniform, 1.5–2.0 × 2.0–3.5 μm		Dong et al. (2019)

**Table:** 

1	Giant cells known	**2**
–	Giant cells unknown	**15**
2	Aborted sporangia known	**3**
–	Aborted sporangia unknown	***G.hydei***
3	Fertile sporangia > 25 μm diameter	**4**
–	Fertile sporangia < 25 μm diameter	**10**
4	Sporangiospores mainly not reniform	**5**
–	Sporangiospores mainly reniform	**7**
5	Columellae mainly ellipsoidal	***G.qichaensis***
–	Columellae mainly not ellipsoidal	**6**
6	Fertile sporangia 14.2–27.1 μm	***G.pingtangensis***
–	Fertile sporangia, 13.0–41.0 μm	***G.lacrispora***
7	Sporangiospores > 4 μm wide	**8**
–	Sporangiospores < 4 μm wide	**10**
8	Sporangiophores branched ≥ 3 times	***G.namwonensis***
–	Sporangiophores branched < 3 times	**9**
9	Columellae mainly globose and subglobose, 4.0–8.0 μm	***G.brasiliensis***
–	Columellae mainly hemispherical, 1.8–4.7 × 2.0–7.7 μm	***G.pamphilae***
10	Apophyses mainly reniform, 2.8–3.5 × 1.8–2.3 μm	***G.reniformis***
–	Apophyses mainly reniform, 3.2–5.5 × 1.7–3.1 μm	***G.apophysata***
11	Sporangiophores branched > 3 times	**12**
–	Sporangiophores branched ≤ 3 times	**14**
12	Giant cells > 6 μm diameter	**13**
–	Giant cells < 6 μm diameter	***G.abortosporangia***
13	Columellae mainly subspherical and ovoid, 2.6–5.2 × 3.2–6.5 μm	***G.oleae***
–	Columellae mainly hemispherical, 4.4–5.6 × 8.5–9.0 μm	***G.multiramosa***
14	Apophyses oval, subglobose and gourd-shaped	***G.bawanglingensis***
–	Apophyses long fusiform, oval and egg-shaped	***G.inconstans***
15	Fertile sporangia > 25 μm diameter	**16**
–	Fertile sporangia < 25 μm diameter	**17**
16	Apophyses vasiform, 5.0–15.0 × 4.5–15.0 μm	***G.pedratalhadensis***
–	Apophyses ellipsoidal to subglobose, 4.5–8.5 × 4.5–6.0 μm	***G.sichuanensis***
17	Columellae hemispherical	**18**
–	Columellae not hemispherical	**19**
18	Apophyses urn-shaped to subglobose, 6.0–12.0 × 6.0–10.0 μm	***G.chlamydospora***
–	Apophyses oval, 7.0–10 × 8.0–8.7 μm	***G.butleri***
19	Sporangiospores ≥ 3.5 μm long	**20**
–	Sporangiospores < 3.5 μm long	**21**
20	Apophyses globose to subglobose, 3.5–6.5 × 3.0–7.0 μm	***G.eborensis***
–	Apophyses pyriform to subglobose, 8.0–12.0 × 7.0–9.5 μm	***G.multispora***
21	Sporangiospores ≥ 4 μm width, mostly not bean-shaped	**22**
–	Sporangiospores < 4 μm width, mostly bean-shaped	**23**
22	Columellae hemispherical, spherical or ovoid, 2.5–12.0 × 2.0–12.0 μm	***G.guangdongensis***
–	Columellae hemispherical and globose, 2.0–3.0 × 3.5–7.0 μm	***G.zunyiensis***
23	Sporangiospores 2.0–3.5 × 2.0–2.5 μm	***G.orasabula***
–	Sporangiospores 1.7–2.1 × 2.1–3.8 μm	***G.koreana***

## ﻿Discussion

Southern China is located in tropical and subtropical areas, which belong to tropical monsoon climate and subtropical monsoon climate. All the samples used in this study were collected from these areas, including Hainan, Sichuan, Yunnan and Guizhou Provinces. This is consistent with the geographical distribution of the species of *Gongronella*, mainly inhabiting tropical and subtropical regions.

The genus *Gongronella* was established in 1952 and its type *Gongronellaurceolifera* was synonymised with *Gongronellabutleri* whose basionym is *Absidiabutleri* (Ribaldi 1952). Numbers of this genus have increased rapidly recently, with as many as 17 species being described between 2015 and 2024 and the genus currently has a total of 25 members including the six new species proposed herein, all of which are listed in Table 3. However, there are no systematic analyses of the morphological characteristics of the species of *Gongronella*. In this study, the morphological characteristics of the 24 species of *Gongronella* were comparatively analysed (Table 4), except *G.banzhaoae*. Since *G.banzhaoae* only has molecular data and no morphological description, it is not compared in this study.

Since 2019, phylogenetic analyses of *Gongronella* have mainly been conducted on the basis of morphological characteristics and ITS+LSU sequence (Zhang et al. 2019). In this study, new TEF, ACT and RPB1 protein-coding sequences were added for the construction of phylogenetic trees and the results were basically consistent with previous studies based on ITS+LSU. Twelve strains were grouped into six individual clades and two strains were grouped along with *G.pamphilae*. Compared with *G.multiramosa*, *G.abortosporangia* has more abundant and various aborted sporangia, smaller fertile sporangia and smaller columellae (Wang et al. 2023a). Compared with *G.pamphilae*, *G.reniformis* has smaller sporangia and sporangiospores, as well as different shapes of columellae and apophyses. Compared with *G.brasiliensis*, *G.reniformis* has smaller sporangiophores, different columella shapes and smaller giant cells (Tibpromma et al. 2017). Compared with *G.zunyiensis*, *G.apophysata* has larger sporangia, as well as different shapes of columellae, apophyses and chlamydospores (Dong et al. 2019). Compared with *G.inconstans*, *G.bawanglingensis* has smaller sporangiospores, larger columellae, different shapes and sizes of apophyses. Compared with *G.qichaensis*, the *G.bawanglingensis* has smaller sporangia, different columellae and apophysis shapes (Wang et al. 2023a). *G.pingtangensis* and *G.namwonensis* are different in size and shape of columellae (hemispherical vs. globose) and apophyses (oval vs. subglobose). *G.namwonensis* has more shapes of giant cells (Crous et al. 2020). These significant morphological differences, coupled with those phylogenetically independent clades, ensure their novelty (Wang et al. 2023a). As for *G.pamphilae*, two strains were grouped into an independent separate clade and there are only two base pairs of difference in ITS rDNA sequences. As no morphological descriptions were provided for *G.pamphilae* in its protologue, we classified the two isolates together as the new record species of *G.pamphilae* only based on molecular phylogenetic analyses.

In summary, the molecular phylogenetic and morphological results support the identification of the six new species for the 12 strains cultured in this study, namely *G.abortosporangia*, *G.reniformis*, *G.apophysata*, *G.bawanglingensis*, *G.pingtangensis*, *G.inconstans* and two strains as new record species of *G.pamphilae*, complementing the morphological description of *G.pamphilae*. TFE, ACT and RPB1 protein-coding sequences were newly added to construct the phylogenetic evolutionary tree and the results were basically consistent with ITS+LSU results. The morphology of members of the genus *Gongronella* was systematically described herein, with a morphological description table being established for the described strains of *Gongronella* and the new strains described in this study (Table 4).

## Supplementary Material

XML Treatment for Gongronella abortosporangia

XML Treatment for Gongronella apophysata

XML Treatment for Gongronella bawanglingensis

XML Treatment for Gongronella inconstans

XML Treatment for Gongronella pamphilae

XML Treatment for Gongronella pingtangensis

XML Treatment for Gongronella reniformis

## ﻿Additional information

### Author ORCIDs

Yi-Xin Wang https://orcid.org/0009-0001-5231-914X

Heng Zhao https://orcid.org/0000-0003-2938-5613

Yang Jiang https://orcid.org/0009-0003-1292-610X

Xin-Ye Liu https://orcid.org/0009-0004-9396-1763

Xiao-Yong Liu https://orcid.org/0000-0002-8808-010X

### Data availability

The sequences were deposited in the GenBank database.

## Supplementary materials

Supplementary material 1 The combined ITS+LSU+TEF+ACT+RPB1 sequence matrix used in this study Data type fas Explanation note The sequence matrix included 43 strains in 25 species of *Gongronella*, with *Cunninghamellaechinulata* CBS 156.28 as outgroup. A total of 4,080 characters comprised ITS rDNA (1–989), LSU rDNA (990–1967), TEF rDNA (1968–2172), ACT rDNA (2173–2948) and RPB1 rDNA (2949–4080). Among them, there were 2866 constant, 562 variable but parsimony non-informative and 652 parsimony informative characters. https://binary.pensoft.net/file/1178978This dataset is made available under the Open Database License (http://opendatacommons.org/licenses/odbl/1.0/). The Open Database License (ODbL) is a license agreement intended to allow users to freely share, modify, and use this Dataset while maintaining this same freedom for others, provided that the original source and author(s) are credited.Yi-Xin Wang
